# Cyclin-dependent kinase 4 and 6 inhibitors and the breast cancer immune ecosystem: immune remodeling, resistance, and therapeutic reprogramming

**DOI:** 10.3389/fimmu.2026.1923129

**Published:** 2026-08-21

**Authors:** Yuhan Tang, Yingze Zhu, Shaochun Liu, Xiaoxi Han, Haolu Zhang, Wenhui Zhao

**Affiliations:** 1 Department of Medical Oncology, Harbin Medical University Cancer Hospital, Harbin Medical University, Harbin, China; 2 Department of General Surgery, West China Ya’an Hospital, Sichuan University, Ya’an, China

**Keywords:** antitumor immunity, biomarkers, breast cancer, cyclin-dependent kinase 4 and 6 inhibitors, drug resistance, ferroptosis, immune checkpoint blockade, nanomedicine

## Abstract

Cyclin-dependent kinase 4 and 6 inhibitors (CDK4/6 inhibitors) combined with endocrine therapy have become a therapeutic backbone for hormone receptor-positive, human epidermal growth factor receptor 2-negative breast cancer, yet durable disease control is frequently limited by intrinsic and acquired resistance. Canonical tumor-cell mechanisms, including retinoblastoma-pathway escape, cyclin E-cyclin-dependent kinase 2 (CDK2) activation, endocrine adaptation, and phosphoinositide 3-kinase (PI3K)-AKT-mechanistic target of rapamycin (mTOR) signaling, explain only part of this failure because they do not fully capture dynamic immune and stromal remodeling. Preclinical and translational studies indicate that early CDK4/6 inhibition can enhance antigen presentation, activate interferon-related programs, restrain regulatory T cells, and promote a T-cell-inflamed state. These effects are conditional and may not persist during prolonged treatment. Sustained therapy can instead drive heterogeneous resistant niches characterized by stromal remodeling, myeloid recruitment, checkpoint adaptation, and T-cell dysfunction. This immune-state dependence provides a rationale for immune checkpoint blockade, although clinical combinations have shown mixed efficacy and clinically relevant hepatic, pulmonary, and hematologic toxicities. Sequential or lead-in strategies therefore warrant prospective evaluation. Oxidative phosphorylation (OXPHOS) and redox adaptation may sustain selected resistant states and expose context-dependent ferroptotic vulnerabilities. Ferroptosis may connect tumor-cell killing with immune regulation, whereas nanomedicine may improve tumor-selective delivery. Both strategies remain largely preclinical and require further evaluation of pharmacokinetics, biodistribution, toxicity, manufacturability, and immune-cell safety. This Review distinguishes intrinsic from acquired resistance across interpatient, intratumoral, spatial, and temporal dimensions. It integrates tumor-cell escape with cytokine, immune, stromal, vascular, and metabolic remodeling and summarizes emerging therapeutic strategies. We further propose a candidate biomarker-informed framework that integrates genomic profiling, spatial immune architecture, circulating biomarkers, T-cell receptor (TCR) dynamics, transcriptomic and single-cell analyses, artificial intelligence (AI)-assisted multimodal integration, and longitudinal sampling. This framework is intended to support biomarker development and prospective trial design rather than current clinical decision-making, providing a translational basis for testing state-informed and sequence-aware therapeutic strategies.

## 1 Introduction

CDK4/6 inhibitors combined with endocrine therapy are a central treatment backbone for hormone receptor-positive, human epidermal growth factor receptor 2-negative (HR+/HER2−) advanced breast cancer (1, 2). Their principal antitumor action is suppression of retinoblastoma protein phosphorylation and G1-S cell-cycle progression (2, 3). Increasing clinical and experimental evidence, however, indicates that CDK4/6 inhibition exerts biological effects beyond tumor-cell cytostasis, particularly within the tumor immune microenvironment (3, 4).

Despite substantial clinical benefit, intrinsic and acquired resistance remain major barriers to durable disease control (5, 6). Sequencing of CDK4/6 inhibitor-exposed tumors has identified candidate resistance alterations involving RB1, AKT1, RAS, AURKA, CCNE2, ERBB2, and FGFR2, together with loss of estrogen-receptor expression (5). Paired circulating tumor DNA (ctDNA) analyses from PALOMA-3 further demonstrated treatment-associated clonal evolution, including emergent RB1 mutations in a minority of palbociclib-treated patients and evolution of PIK3CA- and ESR1-mutant clones (6). The heterogeneity and limited prevalence of individual alterations indicate that established tumor-cell-intrinsic mechanisms explain only part of clinical resistance (5, 6). Consistent with this complexity, OXPHOS dependency has been identified in a subset of endocrine therapy- and palbociclib-resistant ER-positive metastatic breast cancers, linking mitochondrial adaptation to redox regulation and potential immune-ferroptotic vulnerabilities (7). However, this finding remains limited to a subset of tumors and has not been validated as a clinical biomarker or treatment-selection strategy.

In parallel with tumor-cell adaptation, CDK4/6 inhibition can reshape antitumor immunity. Breast-cancer models and serial clinical biopsies showed enhanced tumor-cell antigen presentation and interferon-associated programs together with reduced regulatory T-cell (Treg) proliferation following CDK4/6 blockade (4). Complementary preclinical studies also reported increased effector T-cell activation despite reduced T-cell proliferation after short-term treatment (8). Consistent with these observations, the neoMONARCH study detected immune-response transcriptional changes during short-course abemaciclib therapy (9). Together, these findings support an early immune-priming effect, although its duration and clinical significance remain uncertain.

Longitudinal studies suggest that these early immune changes are not uniformly maintained during treatment. Later or resistant samples showed suppressive myeloid differentiation and mixed cytotoxic-inhibitory immune states (10, 11), whereas CORALLEEN and NeoRHEA reported attenuation of antigen-presenting or tissue-resident-memory features (12, 13). Mechanistic studies further identified CCL2-mediated Treg chemotaxis (14), an IL-17A-secreting γδ T-cell-CX3CR1-positive macrophage circuit (15), and a neutrophil-activating secretome (16). Clinical studies combining CDK4/6 inhibitors with immune checkpoint blockade likewise illustrate why early immune priming cannot be directly equated with clinical benefit. Evidence from abemaciclib plus pembrolizumab (17), the NEWFLAME regimen (18), and ribociclib plus spartalizumab (19) demonstrated heterogeneous efficacy together with distinct toxicity profiles. Collectively, current evidence supports dynamic and context-dependent immune remodeling rather than a universal trajectory of immune activation or suppression, and prospective studies have yet to establish a validated treatment-selection strategy (20).

This Review examines CDK4/6 inhibitor response and resistance as a dynamic process involving tumor-cell adaptation and microenvironmental remodeling. We first summarize the early immune responses induced by CDK4/6 inhibition before discussing intrinsic and acquired resistance across interpatient, intratumoral, spatial, and temporal dimensions. We then integrate tumor-cell resistance mechanisms with immune, stromal, vascular, and metabolic remodeling to define candidate resistance states and therapeutic opportunities. Finally, we discuss emerging immunotherapeutic strategies, ferroptosis, nano-enabled therapies, and a candidate biomarker-informed framework for longitudinal tissue and blood studies. Throughout this Review, these concepts are presented as investigational frameworks rather than clinical recommendations.

## 2 Early immune activation induced by CDK4/6 inhibitors

### 2.1 Tumor-cell antigen presentation and viral-mimicry signaling

Although CDK4/6 inhibitors were originally developed to suppress retinoblastoma-protein phosphorylation and G1-S cell-cycle progression, one of their earliest biological effects is enhanced tumor-cell immunogenicity. The most consistently described mechanisms involve increased antigen presentation, endogenous retroviral or viral-mimicry signaling, and interferon-associated transcriptional responses. In breast-cancer models and serial biopsies, CDK4/6 inhibition reduced E2F-DNMT1 activity, activated endogenous retroviral elements, increased intracellular double-stranded RNA, induced type III interferons, and enhanced antigen-processing and presentation programs (4).

Low-dose palbociclib or abemaciclib increased HLA expression and generated new HLA ligands in breast-cancer cell lines, whereas abemaciclib expanded the MHC-I immunopeptidome in defined breast-cancer models (21, 22). Together, these studies support enhanced tumor-cell antigen presentation as an early pharmacodynamic consequence of CDK4/6 inhibition. However, whether this response consistently translates into antigen-specific T-cell activation or persists during prolonged treatment remains uncertain.

### 2.2 Effector T-cell activation, memory programs, and regulatory T-cell restraint

Beyond tumor-cell antigen presentation, early immune priming also involves coordinated changes in T-cell activation, differentiation, and regulatory T-cell homeostasis. Short-term CDK4/6 inhibition derepressed NFAT-family activity, increased effector T-cell activation, and enhanced tumor infiltration in experimental systems (8). In mouse and human CD8-positive T cells, CDK4/6 inhibition increased memory-precursor features, and separate studies linked short-course treatment to durable endogenous or engineered T-cell memory programs (23, 24). Foundational breast-cancer studies also demonstrated preferential restraint of regulatory T-cell proliferation relative to conventional T-cell compartments (4).

These observations indicate that T-cell activation, proliferation, memory differentiation, and regulatory T-cell restraint represent related but biologically distinct processes. Activated T-cell proliferation can be reduced while cytotoxic activity is retained under defined palbociclib exposures (25). Accordingly, early immune activation may coexist with impaired proliferation, and its presence should not be interpreted as a direct predictor of durable clinical benefit.

### 2.3 Chemokine-mediated recruitment and myeloid-dependent immune support

In addition to tumor-cell and T-cell changes, early immune priming is accompanied by chemokine-mediated immune recruitment and myeloid remodeling. In murine mammary tumors, CDK4/6 inhibition induced tumor-cell CCL5, CXCL9, and CXCL10 and increased recruitment of activated CD8-positive T cells without increasing regulatory T-cell recruitment (26). A separate breast-cancer model linked CDK4/6 inhibition to macrophage reprogramming and enhanced intratumoral CD8-positive T-cell activity through a macrophage migration inhibitory factor-dependent mechanism (27).

Collectively, these studies suggest that early chemokine signaling and myeloid support may reinforce antitumor immunity during the initial treatment phase. Whether these changes are maintained during prolonged treatment or contribute directly to clinical benefit remains to be established.

### 2.4 Checkpoint-ligand regulation and model dependence

Early immune remodeling also extends to immune checkpoint regulation, providing a mechanistic basis for subsequent combination strategies. However, the direction of checkpoint-ligand regulation varies across experimental models. Palbociclib reduced PVR and PD-L1 expression through the pRB-E2F1 axis in triple-negative breast-cancer cells, whereas abemaciclib promoted lysosomal degradation of B7-H4 in other breast-cancer systems (28, 29). Patient profiling and breast-cancer models further suggested that ribociclib and PD-1 blockade influence nonoverlapping effector and memory-associated T-cell programs (20).

These findings indicate that checkpoint modulation is context dependent rather than uniform across CDK4/6 inhibitors or tumor models. Changes in PD-L1 or other checkpoint ligands therefore provide a biological rationale for combination and sequencing studies but should not be regarded as universal predictive biomarkers or evidence of improved response to immune checkpoint blockade.

### 2.5 Translational evidence and limits of early immune priming

Clinical studies provide an opportunity to examine whether these early immune responses can be detected in patients. The neoMONARCH study provides the strongest translational evidence in HR-positive/HER2-negative breast cancer. A two-week abemaciclib-containing lead-in produced profound cell-cycle arrest together with immune-response transcriptional changes in tumor samples (9). These findings support the existence of an early pharmacodynamic immune response.

**Figure 1:**
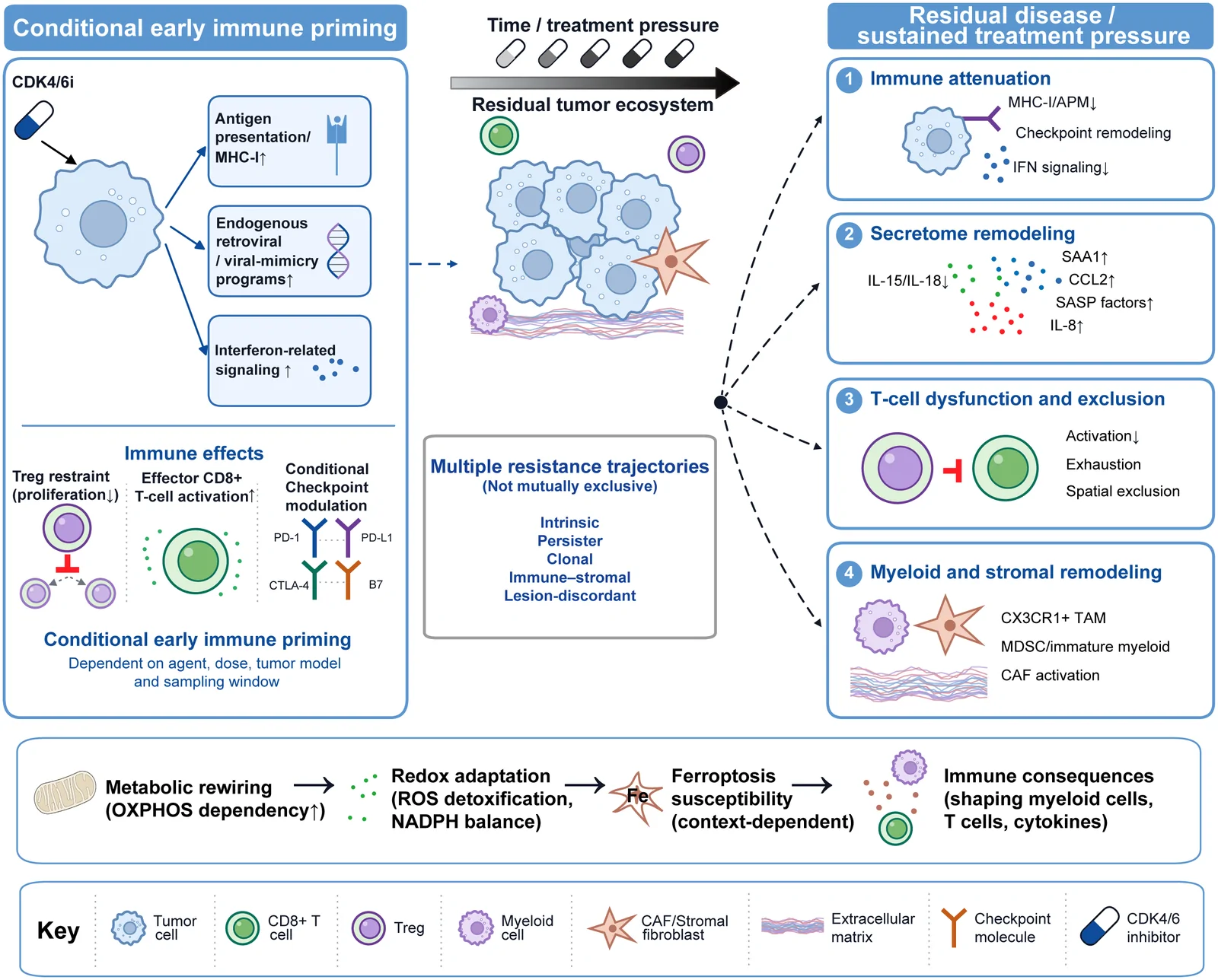
Immune-state transition and ecological remodeling during CDK4/6 inhibitor therapy. CDK4/6 inhibition can initially induce a conditional immune-primed state characterized by increased antigen presentation (MHC-I/APM), endogenous retroviral (ERV)/viral-mimicry programs, interferon-related signaling, restrained Treg proliferation, enhanced effector CD8^+^ T-cell activation, and context-dependent checkpoint modulation. These early immune effects are conditional and transient, varying with the CDK4/6 inhibitor, treatment schedule, endocrine partner, tumor model, and sampling window. Under sustained treatment pressure, the residual tumor ecosystem may evolve into heterogeneous immune-ecological states rather than following an inevitable linear progression. Representative resistant-state features include immune attenuation, secretome remodeling, T-cell dysfunction or spatial exclusion, myeloid and stromal remodeling, and metabolic adaptation linked to OXPHOS dependence, redox homeostasis, and variable ferroptosis susceptibility. Possible trajectories include transient immune priming followed by adaptive persistence, clonal evolution, mixed resistant states, or lesion-discordant immune ecosystems. The illustrated trajectories represent conceptual biological models rather than validated clinical resistance subtypes and require prospective longitudinal human validation.

However, longer treatment durations and different clinical settings have produced non-equivalent findings. CORALLEEN reported treatment- and time-dependent changes in antigen-presenting and immune-cell programs (12), whereas NeoRHEA identified reductions in adaptive and tissue-resident-memory features (13). Matched biopsies obtained during abemaciclib treatment likewise demonstrated heterogeneous interferon, antigen-presentation, T-cell, and myeloid responses (30). Collectively, current evidence suggests that early immune priming represents a transient and context-dependent pharmacodynamic state rather than a stable immune phenotype. Its magnitude and duration vary according to drug, dose, treatment schedule, tumor context, sampled compartment, and treatment window, highlighting that the biological consequences of CDK4/6 inhibition continue to evolve during treatment and ultimately shape the heterogeneous resistance states discussed in the following section (**Figure 1**).

## 3 Resistance to CDK4/6 inhibition: biological origin, heterogeneity, and temporal evolution

### 3.1 A conceptual framework for resistance

Resistance to CDK4/6 inhibition should be viewed as a multidimensional and evolving biological process rather than a simple distinction between early and late treatment failure. Clinical timing defines when resistance becomes apparent, whereas biological origin reflects whether resistance arises from pre-existing tumor characteristics or treatment-driven adaptation. These dimensions are related but not interchangeable: early progression does not necessarily indicate intrinsic resistance, and late progression does not necessarily represent acquired resistance.

**Figure 2:**
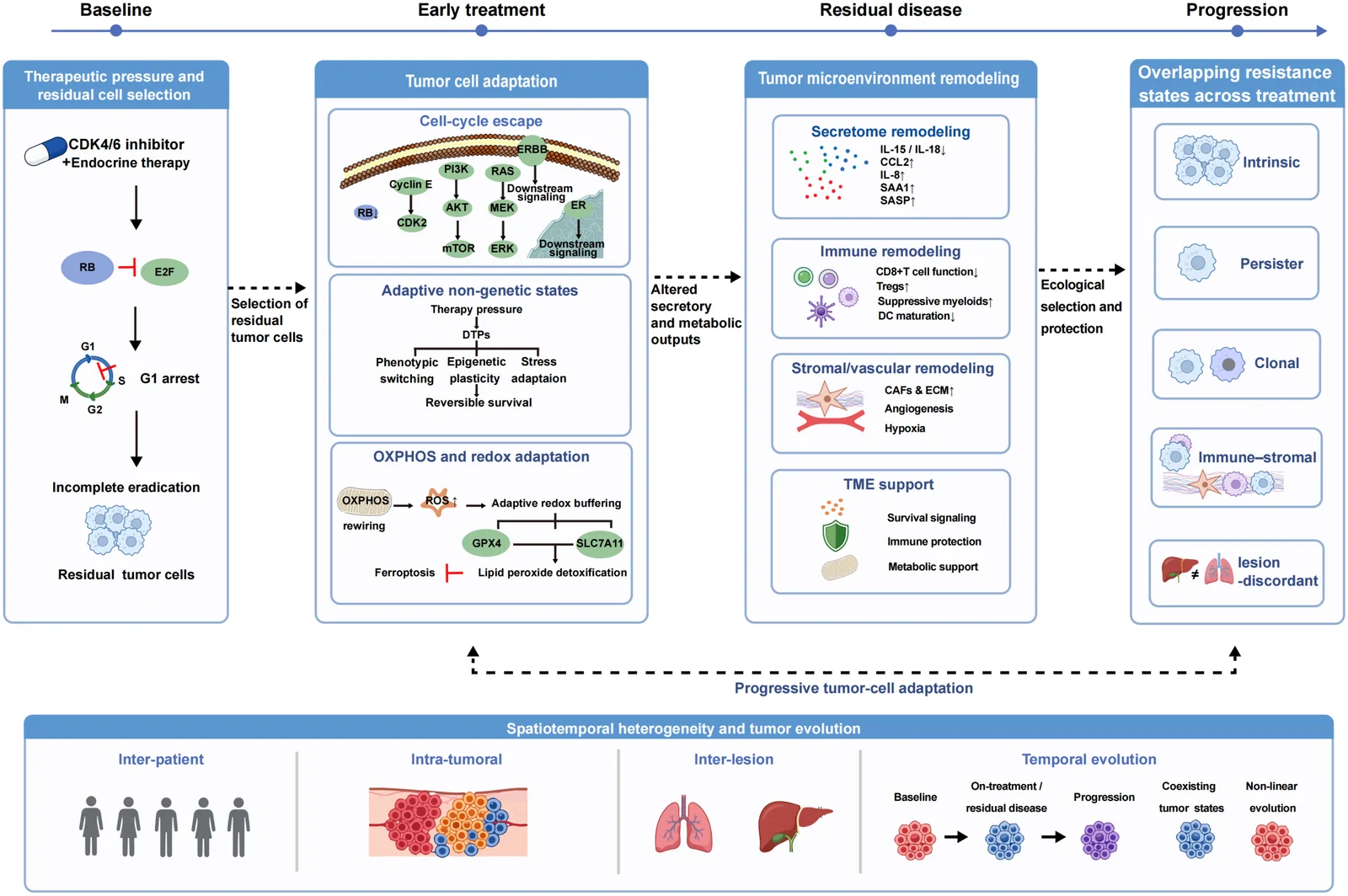
Conceptual framework for the spatiotemporal evolution of tumor-intrinsic and microenvironmental resistance to CDK4/6 inhibitor-based therapy. Baseline and early therapeutic effects. CDK4/6 inhibitor-based therapy suppresses RB phosphorylation, maintains E2F repression, and induces G1 cell-cycle arrest. Incomplete tumor-cell elimination permits the persistence and selection of residual cells that initiate subsequent adaptation. Tumor-cell adaptation. Under therapeutic pressure, residual cells acquire adaptive programs, including cell-cycle escape through compensatory signaling, reversible drug-tolerant persister states driven by phenotypic and epigenetic plasticity, and metabolic adaptation characterized by OXPHOS remodeling and enhanced redox buffering that may reduce ferroptotic susceptibility. Tumor-microenvironment (TME) remodeling. Adaptive tumor cells reshape the microenvironment through altered secretory and metabolic programs, promoting immune suppression, stromal and vascular remodeling, and the establishment of protective niches that support tumor survival, immune evasion, and metabolic adaptation. Heterogeneous resistance states. Continued tumor-cell adaptation and microenvironmental selection contribute to diverse resistance patterns, including intrinsic, persister, clonally acquired, immune-stromal-mediated, and lesion-discordant resistance. These represent overlapping and dynamic biological states rather than fixed clinical subtypes. Spatiotemporal evolution. Resistance develops across multiple dimensions, including inter-patient diversity, intra-tumoral spatial heterogeneity, inter-lesion discordance, and non-linear temporal evolution, with distinct resistance states emerging and coexisting throughout disease progression. Solid arrows indicate relatively direct biological relationships; dashed arrows indicate indirect, context-dependent, or evolutionary transitions; T-bars indicate inhibitory interactions. The illustrated mechanisms and resistance states are representative and are not expected to occur uniformly in every patient.

Accordingly, resistance should be interpreted across complementary dimensions, including biological origin, molecular adaptation, clonal evolution, spatial organization, and temporal dynamics. Under continued therapeutic pressure, residual-cell persistence is followed by tumor-cell adaptation, progressive microenvironmental remodeling, ecological selection, and the emergence of heterogeneous resistance states across patients, metastatic lesions, and treatment stages (**Figure 2**). This evolutionary framework forms the basis for understanding the resistance mechanisms discussed below.

### 3.2 Biological origins and evolutionary mechanisms of resistance

Resistance arises through both pre-existing biological characteristics and treatment-driven evolutionary processes. Intrinsic resistance reflects limited dependence on the CDK4/6-retinoblastoma (RB) pathway before therapy, whereas acquired resistance develops through continuous selection and adaptation under therapeutic pressure.

Intrinsic resistance most commonly results from impaired RB-dependent cell-cycle control or activation of baseline bypass pathways. Canonical CDK4/6 inhibition requires an intact RB checkpoint, and baseline RB1 loss or functional RB deficiency can compromise cell-cycle arrest (5). Cyclin E1/E2-CDK2 activation, alternative CDK6 complexes, and related bypass pathways provide additional proliferative routes (31–33). RB1-deficient models have also identified PRMT5 as a potential therapeutic vulnerability, although RB1 loss does not uniformly predict PRMT5 dependence (34). Importantly, genomic RB1 alterations do not necessarily reflect functional RB deficiency, and genomic, protein, phosphorylation, and transcriptional analyses should therefore be regarded as complementary assessments of RB pathway activity.

Even in tumors with preserved RB signaling, baseline oncogenic and lineage programs strongly influence treatment sensitivity. Activation of PI3K-AKT-mTOR or RAS-MAPK signaling, PTEN loss, FGFR or ERBB signaling, MYC activation, ESR1 mutation, reduced luminal differentiation, and diminished endocrine dependence can all reduce responsiveness to CDK4/6 inhibition (5, 31, 35). Likewise, pre-existing immune exclusion, suppressive myeloid populations, and fibroblast- or extracellular matrix-mediated protection may restrict the conversion of tumor-cell arrest into durable antitumor immunity. Together, these baseline molecular and ecological characteristics determine the biological context in which CDK4/6 inhibition is initiated.

Following an initial response, resistance evolves through continued selection under therapeutic pressure rather than through acquisition of a single dominant mutation. Longitudinal tissue and plasma analyses have identified treatment-associated alterations involving RB1, ESR1, PIK3CA, AKT, RAS, AURKA, CCNE2, ERBB2, and FGFR-related pathways, although their prevalence and functional significance differ across studies (5, 6). In PALOMA-3, serial circulating tumor DNA analyses demonstrated emergent RB1 mutations together with expansion of ESR1- and PIK3CA-mutant clones during palbociclib treatment (6). Similarly, serial single-cell profiling in FELINE revealed distinct patient-specific subclonal trajectories that converged on common proliferative and growth-factor signaling programs despite different evolutionary origins (36). These findings indicate that resistance commonly reflects continued evolutionary diversification rather than linear acquisition of a single resistance mechanism.

Selection is accompanied by adaptive biological reprogramming. Cyclin E-CDK2 activation, PI3K-AKT-mTOR feedback, MAPK reactivation, receptor tyrosine kinase compensation, altered estrogen-receptor signaling, chromatin remodeling, transcriptional plasticity, and drug-tolerant persister states enable genetically related cells to occupy distinct functional states during treatment (5, 7, 31–36). Context-dependent reliance on oxidative phosphorylation (OXPHOS) in subsets of endocrine therapy- and palbociclib-resistant ER-positive tumors further illustrates how metabolic adaptation can coexist with genomic evolution (7).

As treatment continues, resistance extends beyond malignant cells to the surrounding microenvironment. Longitudinal studies have documented dynamic immune and stromal remodeling during CDK4/6 inhibitor treatment (10, 11), while mechanistic studies have implicated CCL2-mediated regulatory T-cell recruitment (14), senescence-associated neutrophil activation (16), stromal senescence (37), CD63-positive fibroblast-derived exosomal signaling (38), and fibroblast-tumor mutualism (39) as contributors to adaptive resistance. Although much of this evidence remains preclinical, these observations collectively indicate that acquired resistance reflects coordinated evolution of tumor cells and the surrounding microenvironment rather than isolated genomic alterations alone. These evolutionary processes do not occur uniformly but generate substantial biological diversity across patients, lesions, and treatment stages.

### 3.3 Multidimensional heterogeneity

The evolutionary routes leading to resistance vary substantially across patients. Endocrine sensitivity, prior treatment exposure, metastatic burden, and host immunity all influence resistance development. Genomic profiling has demonstrated variable frequencies of RB1, ESR1, PIK3CA, AKT1, and PTEN alterations across clinical settings (5, 6, 32), while transcriptomic subtype, luminal differentiation, immune infiltration, stromal composition, and metabolic state further diversify treatment responses (40). Consistent with this variability, neoMONARCH and CORALLEEN reported distinct pharmacodynamic and immune responses despite broadly similar therapeutic strategies (9, 12). Together, these findings indicate that no single molecular alteration or immune feature adequately explains clinical resistance.

Heterogeneity also develops within individual tumors and across metastatic lesions. Distinct subclones may harbor different genomic alterations while simultaneously occupying diverse transcriptional and functional states shaped by immune, stromal, vascular, hypoxic, and metabolic niches. Serial single-cell analyses demonstrated divergent evolutionary trajectories that converged on common resistant phenotypes (36). Imaging mass cytometry likewise identified reduced immune-cell abundance together with increased myeloid representation in metastatic lesions (41). These observations indicate that spatial heterogeneity reflects both clonal diversity and local microenvironmental adaptation.

Resistance continues to evolve throughout treatment. Pretreatment samples define baseline molecular and immune states, early on-treatment biopsies capture pharmacodynamic responses, whereas residual disease and progression samples reveal clonal selection, adaptive signaling, and progressive microenvironmental remodeling. Longitudinal analyses from PALOMA-3 demonstrated evolving ctDNA profiles during therapy (6), while serial tumor studies documented progressive immune remodeling during ribociclib treatment (10, 11, 30). Single-cell profiling further revealed convergent resistant phenotypes despite distinct evolutionary trajectories (36). Together, these findings emphasize that resistance should be interpreted as a continuously evolving biological process rather than a static endpoint.

Because biological origin, spatial organization, and temporal evolution cannot be captured by a single specimen, tissue biopsy, ctDNA, and complementary molecular profiling should be regarded as complementary rather than interchangeable approaches. Integrating these dimensions provides the biological framework for understanding how resistance evolution is accompanied by dynamic remodeling of immune and stromal cell populations, which are examined in the following section.

## 4 Immune-cell and stromal populations shaping CDK4/6 inhibitor response and resistance

### 4.1 CD8-positive effector and memory T cells

Among the immune populations altered during CDK4/6 inhibitor treatment, CD8-positive T cells most clearly illustrate the distinction between proliferation, cytotoxic function, and memory differentiation. CDK4/6 inhibition can restrict activated T-cell proliferation while preserving selected effector and memory programs. In breast-cancer models, abemaciclib induced a T-cell-inflamed transcriptional state, increased tumor T-cell infiltration, enhanced the activity of PD-L1 blockade, and supported immune memory upon rechallenge (42).

Exploratory analyses of two small phase I studies of palbociclib, pembrolizumab, and letrozole associated higher baseline effector-memory CD4-positive and CD8-positive populations, including selected KLRG1-positive and ICOS-positive subsets, with response. By contrast, tumor-infiltrating lymphocytes, tumor mutational burden, and PD-L1 showed no comparable association (44). RIBECCA similarly reported increased adaptive-immune signatures and reduced Tregs, but only limited changes in effector- and memory-T-cell populations. Increased T-cell clonality was observed in selected patients rather than as broad clonal expansion (45).

Later treatment stages further illustrate the dynamic nature of CD8-positive T-cell responses. Although abemaciclib plus PD-1 blockade increased effector CD8-positive T cells in brain-metastasis models (46), NeoRHEA reported reduced CD8-positive/CD103-positive tissue-resident-memory features after prolonged therapy (13). Serial tumor analyses also identified diminished T-cell activation and recruitment during ribociclib resistance (10), whereas late-progressing tumors retained cytotoxic lymphocytes despite inhibitory SPP1-CD44 and MDK-NCL interactions (11). CD8-positive T-cell abundance therefore does not necessarily indicate sustained cytotoxic function, which also depends on activation state, exhaustion, spatial organization, and cell-cell interactions.

### 4.2 Regulatory T cells

Tregs provide a clear example of a population whose early pharmacodynamic behavior may differ from its role in resistant tissue. Foundational models demonstrated direct suppression of Treg proliferation (4), while two human studies reported reductions in circulating Tregs during treatment (43, 45). Lower Treg frequencies were associated with response in both studies, with the most pronounced change observed in selected effector-Treg populations (43, 45). These early pharmacodynamic effects, however, do not preclude later Treg persistence or recruitment within resistant tumors. Early restraint of Treg proliferation may reflect a direct, compartment- and timing-dependent pharmacological effect, whereas later enrichment may arise from compensatory chemokine networks, stromal remodeling, or selective persistence of suppressive populations. In experimental HR-positive/HER2-negative systems, palbociclib plus fulvestrant induced a senescence-associated CCL2 program that promoted Treg chemotaxis (14), and brain-metastasis models further showed that Treg changes depended on abemaciclib and PD-1 blockade within a site-specific microenvironment (46). Circulating Treg reduction should therefore not be equated with sustained intratumoral depletion.

### 4.3 Dendritic cells and other antigen-presenting myeloid populations

Dendritic-cell responses appear particularly sensitive to tissue compartment and treatment timing. In a small phase I combination-study analysis, palbociclib-containing therapy altered circulating dendritic-cell composition, increased the proportion and CD83 expression of conventional type 1 dendritic cells, and increased HLA-DR expression on classical monocytes (44). These findings are compatible with peripheral maturation but do not establish intratumoral cDC1 expansion or isolate the contribution of individual agents. By comparison, tumor-based studies have produced different, but not necessarily contradictory, observations. CORALLEEN detected reductions in selected antigen-presenting and innate-immune signatures after ribociclib plus letrozole, whereas matched abemaciclib-treated biopsies showed heterogeneous increases in antigen-presentation programs in macrophages and dendritic cells (12, 30). Differences in tissue versus blood, treatment window, regimen, and residual-tumor selection may account for part of this variation. Accordingly, DC frequency, CD83 expression, HLA-DR expression, antigen-presenting-cell signatures, and DC-CD8 spatial proximity should be regarded as complementary rather than interchangeable biological readouts.

### 4.4 MDSCs, monocytes, neutrophils, and IL-17-associated myeloid circuits

Myeloid-derived suppressor cells, immature myeloid cells, monocytes, neutrophils, and macrophages represent distinct but interacting populations and should not be inferred from a single lineage marker. In patients with metastatic HR-positive/HER2-negative breast cancer, circulating monocytic and polymorphonuclear MDSCs decreased during treatment, whereas a palbociclib-based combination study reported fewer classical monocytes but higher monocyte HLA-DR expression (43, 44). By contrast, tumor-based studies identified locally suppressive myeloid states during resistance. Single-cell profiling in a HER2-positive model revealed an immunosuppressive immature-myeloid-cell population in rapidly resistant tumors (47), while serial ribociclib-treated human tumors linked suppressive myeloid differentiation to impaired IL-15/IL-18-related myeloid-CD8 communication (10), indicating that circulating abundance and intratumoral functional state may change independently. Additional studies further suggest context-dependent neutrophil and IL-17-associated remodeling. A palbociclib-induced secretome enriched in IL-8 and serum amyloid A1 recruited and activated neutrophils *in vitro* (16), whereas CDK4/6 inhibition induced a hypoxia-sensitive CCL2 circuit that recruited IL-17A-producing γδT cells and promoted suppressive CX3CR1-positive macrophage remodeling in HR-positive/HER2-negative mammary tumors (15). Together, these findings support context-dependent myeloid remodeling but do not define a universal neutrophil, IL-17, or MDSC-driven resistance state.

### 4.5 Tumor-associated macrophages

Tumor-associated macrophage responses to CDK4/6 inhibition are highly context dependent and are shaped by tumor-cell, fibroblast, cytokine, and anatomical-site signals. In HR-positive/HER2-negative models, palbociclib-induced fibroblast senescence increased IGF1 and FGF7 secretion, promoted a STAT3-Tyr705/ARG1-associated macrophage state, depleted extracellular arginine, and reduced lymphocyte viability. CSF1R inhibition attenuated this CAF-TAM-lymphocyte circuit and enhanced palbociclib activity (48).

Other studies indicate that CDK4/6 inhibition can support either macrophage-mediated antitumor activity or suppressive macrophage states. Increased phagocytosis was observed with CD47 blockade (49), whereas RANK-pathway and IL25-related models implicated macrophages in immune suppression and resistance (50, 51). Macrophage-state changes were also reported in radiotherapy and immune-checkpoint combinations, although the specific contribution of CDK4/6 inhibition could not be isolated (52). These findings emphasize that macrophage abundance should be distinguished from functional state, spatial organization, and interactions with fibroblasts and lymphocytes. Broader spatial studies have identified fibroblast-associated and SPP1- or CD163-enriched macrophage niches, but these associations were described in broader breast-cancer settings and are not specific to CDK4/6 inhibitor resistance (53–56).

### 4.6 Cancer-associated fibroblasts and therapy-induced stromal senescence

Cancer-associated fibroblasts and therapy-induced senescent stromal cells can translate stromal cell-cycle arrest into growth-factor, cytokine, extracellular-vesicle, and matrix-remodeling signals. Prolonged palbociclib exposure induced fibroblast senescence, an NF-κB-dependent senescence-associated secretory phenotype, Gr-1-positive myeloid recruitment, and enhanced tumor growth in preclinical models (57). Related studies linked senescent fibroblast-derived growth factors and CXCL1- or STAT3-associated stromal programs to reduced sensitivity to CDK4/6 inhibition (48, 58, 59).

Fibroblasts may also reinforce tumor-cell resistance through extracellular-vesicle, growth-factor, and reciprocal receptor-signaling programs. Patient-linked and experimental studies show that these signals can sustain resistant tumor-cell survival and proliferation (38, 39). Stromal senescence has also been associated with metastatic seeding, while treatment sequence influenced senescent-cell accumulation and therapeutic outcome in radiotherapy-combination models (37, 60). However, CAFs should not be regarded as a uniform immunosuppressive population. Broader spatial studies have identified functionally distinct fibroblast states associated with extracellular-matrix and vascular remodeling that may restrict immune-cell access (61–65).

### 4.7 Population-level synthesis: timing, location, and resistance context

Collectively, these observations show that cellular abundance alone is insufficient to define immune function during CDK4/6 inhibitor treatment. The biological significance of each lymphoid, myeloid, and stromal population depends on treatment phase, anatomical compartment, functional state, spatial organization, and intercellular interactions. Peripheral-blood changes should not be assumed to represent intratumoral states, and early pharmacodynamic changes may differ from those present in residual or progressing disease. T-cell subsets, Treg frequency, dendritic-cell maturation markers, myeloid states, macrophage phenotypes, and fibroblast-associated niches should therefore be interpreted as complementary research readouts rather than interchangeable or validated treatment-selection biomarkers. These population-level patterns and their principal evidence boundaries are summarized in **Table 1** and incorporated into the resistance framework shown in **Figure 2**.

**Table 1:** Immune mechanisms regulated by CDK4/6 inhibitors and associated resistance states in breast cancer.

Mechanism	CDK4/6 inhibitors agent	Model or evidence source	Immune component	Main finding	Evidence level	Interpretation limit	Ref No.
I. Tumor antigen presentation							
RB-E2F-DNMT1 viral mimicry, antigen presentation and Treg restraint	Selective CDK4/6 inhibitors (multiple study compounds)	Breast-carcinoma and other tumor models; serial breast-cancer biopsies	Tumor antigen presentation; Treg	CDK4/6 inhibition induced ERV/dsRNA-type III interferon and antigen-presentation programs and restrained Treg proliferation in defined models and serial breast-cancer biopsies.	Preclinical plus translational	Foundational early-priming evidence; it does not show that acquired RB1 loss reverses these programs or that they persist during resistance.	(4)
HLA ligand induction	Palbociclib; abemaciclib	Breast-cancer cell lines; immunopeptidomics	HLA/MHC ligands	Low-dose treatment increased HLA expression and induced pathway-related HLA ligands	Cell line	Antigen-specific T-cell responses and patient benefit were not established	(21)
Rb-dependent immunopeptidome remodeling	Abemaciclib	HR-positive and triple-negative breast-cancer cells; multi-omics	MHC-I immunopeptidome	Expanded MHC-I peptide presentation through Rb-dependent chromatin and transcriptional remodeling	Cell line/multi-omics	Cell-based multi-omics evidence; clinical antigen-specific immunity and acquired-RB1-loss effects remain untested.	(22)
Cyclin E-associated interferon change	Palbociclib resistance	ER-positive resistant cell lines and oncolytic adenovirus assays	Type I interferon signaling	Cyclin E-high resistant cells showed reduced type I interferon signaling and altered viral susceptibility	Cell line	Direct but virotherapy-specific; no patient immune-cold phenotype	(68)
II. T-cell activation and regulation							
Effector T-cell activation	Small-molecule CDK4/6 inhibitors (multiple)	Ex vivo spheroids and murine syngeneic models	Effector T cells; NFAT	Short exposure increased T-cell activation and tumor infiltration despite reduced proliferation	Animal model/ex vivo	Pan-cancer extrapolated evidence; not direct breast-cancer clinical proof	(8)
Chemokine-mediated CD8 recruitment	CDK4/6 inhibitor (study compound)	Murine mammary-tumor models	CCL5; CXCL9; CXCL10; CD8 T cells	Tumor-cell stress induced chemokines and recruitment of activated CD8 T cells without increased Treg recruitment	Animal model	Demonstrated in murine models; agent and human relevance require validation	(26)
CD8 memory-precursor formation	Palbociclib or abemaciclib in patient profiling	Mouse/human CD8 T cells; breast-cancer patient samples	CD8 memory precursors; MXD4-MYC	CDK4/6 inhibition shifted activated CD8 T cells toward memory-precursor features	Translational	Immune phenotype does not establish improved clinical outcome	(23, 24)
Reduced tissue-resident memory features	Palbociclib plus endocrine therapy	NeoRHEA phase 2 on-treatment tumors	CD8-positive CD103-positive Trm cells	Treatment reduced tissue-resident memory marker features	Clinical/translational	Single-arm early-breast-cancer study; not acquired-resistance proof	(13)
MAPK/SASP-CCL2-Treg recruitment	Palbociclib plus fulvestrant	Experimental HR+/HER2- breast-cancer systems	Treg; CCL2; SASP	Treatment induced a SASP/MAPK-linked CCL2 program that promoted Treg chemotaxis	Preclinical	Preclinical HR+/HER2- evidence; patient-level resistance causality and intervention benefit are unproven.	(14)
Mixed cytotoxic and inhibitory late-progression state	CDK4/6 inhibitors plus endocrine therapy	Baseline/progression metastatic single-cell samples	CD8 T cells; NK cells; inhibitory ligand-receptor interactions	Late progression retained cytotoxic activity alongside SPP1-CD44 and MDK-NCL inhibitory interactions	Clinical/translational	Small heterogeneous cohort; does not define a universal exhausted phenotype	(11)
III. Myeloid populations							
Macrophage-supported CD8 immunity	CDK4/6 inhibitor (study compound)	Breast-cancer animal models	MIF axis; TAMs; CD8 T cells	MIF-dependent macrophage reprogramming increased intratumoral CD8 T-cell activity	Animal model	Preclinical and mechanism-specific; not evidence of uniform macrophage polarization in patients	(27)
Longitudinal suppressive myeloid differentiation	Ribociclib plus letrozole	Serial biopsies from high-risk ER-positive breast tumors	Myeloid cells; CD8 T cells; IL-15/IL-18	Tumors growing on treatment showed suppressive myeloid differentiation, impaired myeloid-CD8 communication, and later reduced T-cell activation/recruitment	Translational	Observed in a defined neoadjuvant cohort; not a universal trajectory	(10)
Mixed lymphoid and myeloid adaptation	Abemaciclib plus endocrine therapy	Thirteen matched advanced ER+/HER2- biopsies	T cells; TAMs; dendritic cells	T-cell interferon programs decreased while myeloid antigen-presentation and TREM2-positive TAM changes were heterogeneous	Translational	Small matched cohort; direction differed by immune compartment	(30)
CCL2-IL-17A-TAM resistance circuit	CDK4/6 inhibition	Mouse HR+/HER2- tumors plus paired/correlative human samples	Gamma-delta T cells; IL-17A; CX3CR1-positive TAMs	A CCL2-dependent circuit recruited IL-17A-producing gamma-delta T cells and induced suppressive TAM polarization associated with resistance	Translational	Causal evidence is from mice; human evidence supports association and temporal relevance	(15)
IV. Stromal and vascular remodeling							
Host stromal senescence and metastatic niche	Palbociclib pretreatment	Mouse lung metastatic-niche model	Stroma; macrophage/monocyte programs	Host senescence altered lung immune programs and increased seeding by resistant mammary cells	Animal model	Host-pretreatment model; not direct evidence from treated human metastases	(37)
CAF exosomal miR-20-mediated RB1 suppression	CDK4/6 inhibitors	Resistant ER-positive tumors, cell systems and xenografts	CD63-positive CAFs; exosomal miR-20	CAF-derived miR-20 reduced RB1 and sensitivity to CDK4/6 inhibitors	Translational/preclinical	Stromal resistance mechanism; it does not establish a uniform immune-suppressive CAF state.	(38)
Cancer-fibroblast TGF-beta/ERBB mutualism	Endocrine therapy plus CDK4/6 inhibitors	Serial patient biopsies and perturbation models	Fibroblasts; TGF-beta; ERBB ligands	Treatment-associated reciprocal signaling sustained resistant cancer-cell proliferation	Translational	Direct stromal-growth crosstalk; immune-cell consequences were not measured.	(39)
V. Immune checkpoint modulation							
RB-E2F regulation of PVR and PD-L1	Palbociclib	Triple-negative breast-cancer cells	PVR; PD-L1; pRB-E2F1	Reduced PVR and PD-L1 expression through the pRB-E2F1 axis	Cell line	TNBC cell-line evidence; direction is agent-, pathway-, and model-specific and does not establish improved ICB response.	(28)
B7-H4 degradation	Abemaciclib	Breast-cancer cells and murine tumor models	B7-H4; T cells	Promoted lysosomal B7-H4 degradation and mitigated B7-H4-mediated immune suppression	Animal model/cell line	Preclinical evidence; not a validated patient-selection strategy	(29)
Nonoverlapping T-cell programs with PD-1 blockade	Ribociclib plus PD-1 blockade	Mixed breast/ovarian patient profiling; breast and melanoma mouse models	T-cell effector expansion; memory	CDK4/6 inhibition and PD-1 blockade affected complementary T-cell programs	Translational plus animal model	Mixed-cancer cohort and preclinical sequencing efficacy; extrapolated evidence for clinical sequence	(20)
VI. Translational implications							
Early immune-response transcriptional change	Abemaciclib with or without anastrozole lead-in	neoMONARCH neoadjuvant HR+/HER2- breast tumors	Immune-response gene programs	Two-week abemaciclib-containing treatment produced immune-response transcriptional changes alongside cell-cycle arrest	Translational clinical study	Pharmacodynamic signal; does not prove durable T-cell inflammation or immune-mediated efficacy	(9)
Cyclin E-CDK2 proliferative bypass	Palbociclib	Resistant breast-cancer cells and progression samples	Not directly measured	Cyclin E1/CDK2 perturbation restored sensitivity in a PI3K/mTOR-supported resistant state	Translational/preclinical	Resistance evidence without a direct immune endpoint	(31, 67, 69, 70)
PTEN neddylation and cytokine output	Palbociclib resistance	Luminal breast-cancer models and patient-associated data	Cytokines; chemokines	PTEN neddylation activated PI3K-AKT/AP-1-JUND and increased cytokine/chemokine release	Translational/preclinical	Inflammatory immune-cell remodeling was proposed, not directly shown	(71)
ESR1-mutant CDK4/6 inhibitors resistance	Palbociclib	Real-world sequencing; engineered ER-positive models	Not directly measured	ESR1 Y537S/D538G conferred resistance	Translational/preclinical	No direct immune-remodeling endpoint; evidence gap	(35)

Mechanisms are grouped by their predominant immunological function and may participate in multiple biological processes. Evidence level reflects the highest level of supporting evidence, whereas Interpretation limit summarizes the main caveat for interpreting the available data.

AKT, protein kinase B; AP-1, activator protein 1; CAF, cancer-associated fibroblast; CDK, cyclin-dependent kinase; CX3CR1, C-X3-C motif chemokine receptor 1; dsRNA, double-stranded RNA; ER, estrogen receptor; ERBB, erythroblastic oncogene B receptor; ERV, endogenous retrovirus; FGFR, fibroblast growth factor receptor; HLA, human leukocyte antigen; HR+/HER2−, hormone receptor-positive/human epidermal growth factor receptor 2-negative; ICB, immune checkpoint blockade; IL, interleukin; MHC-I, major histocompatibility complex class I; MIF, macrophage migration inhibitory factor; MYC, MYC proto-oncogene; NFAT, nuclear factor of activated T cells; NK, natural killer; PALOMA-3, PALbociclib Ongoing Trials in the Management of Breast Cancer-3; PD-L1, programmed death ligand 1; PI3K, phosphoinositide 3-kinase; PRMT5, protein arginine methyltransferase 5; PTEN, phosphatase and tensin homolog; RB, retinoblastoma; SASP, senescence-associated secretory phenotype; TAM, tumor-associated macrophage; TGF-β, transforming growth factor beta; TNBC, triple-negative breast cancer; Treg, regulatory T cell; TREM2, triggering receptor expressed on myeloid cells 2.

These population-level observations ultimately require mechanistic interpretation. Defining the tumor-cell pathways and cross-compartment signaling networks that initiate and reinforce immune and stromal remodeling will therefore be essential for understanding resistance evolution, as discussed in the following section.

## 5 Crosstalk between canonical resistance mechanisms and immune remodeling

### 5.1 RB-E2F signaling and antigen-presentation programs

Building on the population-level changes described above, the RB-E2F axis provides the clearest mechanistic link between CDK4/6 inhibition and tumor-cell immune-related transcriptional programs. These early tumor-cell transcriptional changes are partly mediated by RB-dependent suppression of E2F-DNMT1 activity, which enhances endogenous-retroviral, interferon-related, and antigen-presentation programs (4). RB loss or functional inactivation during resistance may therefore reconfigure these outputs.

In TNBC cells, RB depletion increased E2F1-dependent PVR and PD-L1 expression, whereas palbociclib reduced both checkpoint ligands (28). In resistant HR-positive and TNBC models, CDK7 inhibition attenuated E2F-driven transcriptional amplification and enhanced immune-related signaling when combined with CDK4/6 inhibition (66). These findings support a mechanistic connection between RB-E2F signaling and immune-related transcription. Whether acquired RB1 loss consistently reverses early immune priming in HR-positive/HER2-negative tumors remains unclear.

### 5.2 Cyclin E-CDK2 bypass and unresolved immune consequences

Beyond mediating proliferative escape, cyclin E-CDK2 activation may also influence interferon-related transcription. CCNE2 alterations and high cyclin E1 expression have been associated with reduced sensitivity or benefit from CDK4/6 inhibition, whereas genetic suppression of cyclin E1 or CDK2 restored palbociclib sensitivity in resistant models (5, 31, 67). Reduced type I interferon signaling has been observed in cyclin E-high resistant cells, and CDK2-targeted strategies altered interferon-related transcription in selected experimental models (68–70). These findings suggest that cyclin E-CDK2 bypass may extend beyond cell-cycle control to influence immune-related transcription; however, its effects on immune-cell recruitment, effector function, and spatial organization remain largely undefined.

### 5.3 PI3K-AKT-mTOR/PTEN signaling and endocrine-pathway escape

PI3K-AKT-mTOR activation and PTEN dysregulation may connect proliferative escape with secretory remodeling, although direct immune evidence remains limited. In palbociclib-resistant cells, PI3K-mTOR activation sustained cyclin D1 and CDK4 translation, whereas PTEN neddylation activated PI3K-AKT and AP-1/JUND signaling and increased cytokine and chemokine production (31, 71). However, neither study established a defined downstream immune-cell consequence. The immune link is even less clear for endocrine-pathway escape. Although ESR1 Y537S and D538G mutations confer endocrine and palbociclib resistance, no mutation-specific tumor-cell output has been directly linked to a defined immune-microenvironmental phenotype (35). Broader associations between ER signaling and immune-cell biology are not specific to ESR1-mutant resistance (72), and treatment-associated immune changes cannot be attributed directly to ESR1 alterations (13). Current evidence therefore supports PI3K/PTEN and ESR1 as established resistance pathways, whereas their contributions to immune remodeling remain largely indirect.

### 5.4 Stromal reinforcement of tumor-cell resistance and immune remodeling

Stromal signaling reinforces tumor-cell resistance through reciprocal interactions involving RB, AKT, growth-factor signaling, and therapy-induced senescence. CAF-derived exosomal miR-20 reduced RB1 expression, reciprocal TGF-β and ERBB-family signaling sustained resistant proliferation, and a RUNX1-PDGF-BB circuit activated AKT while limiting senescence (38, 39, 73). Among these mechanisms, senescence-associated CCL2 production followed by Treg recruitment provides the clearest direct link between stromal remodeling and immune regulation (14). CDK4/6 inhibitor-induced senescence also exhibited greater immunogenicity than senescence induced by DNA-damaging agents in selected models (74). In contrast, evidence supporting fibroblast-derived secretory factors, extracellular vesicles, and matrix remodeling is based largely on parallel stromal and immune observations rather than direct causal demonstration. Cell-cycle arrest alone should therefore not be interpreted as evidence of an immunosuppressive senescent state.

### 5.5 OXPHOS dependency, redox adaptation, and immune-ferroptotic crosstalk

OXPHOS and redox adaptation represent candidate links between tumor-cell persistence and immune regulation. Increased OXPHOS dependence has been identified in a subset of endocrine therapy- and palbociclib-resistant ER-positive metastatic breast cancers, and inhibition of mitochondrial respiration reduced tumor growth in selected experimental models (7). A complementary immune-to-metabolic connection is provided by T-cell-derived interferon-γ, which represses SLC3A2 and SLC7A11 and increases tumor-cell susceptibility to lipid peroxidation and ferroptosis in experimental systems (75, 76). However, these relationships have not been causally demonstrated in OXPHOS-dependent, CDK4/6 inhibitor-resistant HR-positive breast cancer. Tumor-cell metabolic vulnerability should therefore not be assumed to confer favorable immune consequences, and tumor-cell and immune-cell metabolic states require separate evaluation. Accordingly, OXPHOS dependence should not be considered synonymous with ferroptosis sensitivity.

Overall, the mechanisms discussed in this section differ substantially in both their biological function and the strength of evidence linking them to immune remodeling (**Figure 2**). Consequently, these pathways should not be viewed as interchangeable therapeutic targets but as biologically distinct contexts that may require different immune-reprogramming strategies.

## 6 Immune-reprogramming strategies combined with CDK4/6 inhibition

### 6.1 Principles for immune-state- and sequence-aware combinations

The immune effects of CDK4/6 inhibition provide a biological rationale for therapeutic combinations beyond PD-1/PD-L1 blockade. However, different resistance states are unlikely to benefit from the same intervention. Tumors may remain unresponsive because of impaired antigen presentation or priming, T-cell exclusion, checkpoint-restrained inflammation, dominant myeloid suppression, stromal barriers, or metabolic adaptation. Combination strategies should therefore be matched to the dominant biological limitation. Checkpoint blockade may be most appropriate when pre-existing antitumor immunity is present but restrained, whereas antigen-priming approaches, T-cell-directed therapies, and myeloid or stromal reprogramming may be more relevant when the principal deficit lies in priming, effector-cell function, or microenvironmental suppression. These categories represent biologically informed hypotheses rather than validated treatment-selection rules.

Treatment sequence should also be regarded as a pharmacodynamic variable rather than merely a scheduling consideration. A short CDK4/6 inhibitor lead-in may transiently enhance antigen presentation, interferon-related signaling, and chemokine production, whereas prolonged exposure may favor myeloid accumulation, stromal adaptation, or senescence-associated remodeling. Conversely, tumors dominated by stromal or myeloid suppression may require microenvironmental reprogramming before T-cell-directed intervention.

### 6.2 Immune checkpoint blockade: the most clinically tested but unresolved strategy

Immune checkpoint blockade (ICB) remains the most extensively investigated immunotherapeutic partner for CDK4/6 inhibitors because CDK4/6 inhibition can enhance antigen presentation and promote effector T-cell activity (4, 8). Nevertheless, these biological effects have not consistently translated into durable clinical benefit.

Early clinical studies illustrate both the promise and the limitations of this strategy. Abemaciclib combined with pembrolizumab produced objective responses in metastatic HR+/HER2− breast cancer but was associated with substantial hepatotoxicity, interstitial lung disease or pneumonitis, and treatment-related deaths (17). Similar safety concerns were reported in the NEWFLAME study (18). Ribociclib plus spartalizumab showed limited clinical activity despite increased circulating CD8-positive T cells, while PD-L1 expression, tumor-infiltrating lymphocytes, and tumor mutational burden failed to predict outcome (19). In the PACE trial, adding avelumab to fulvestrant and palbociclib numerically prolonged progression-free survival after prior CDK4/6 inhibition, although the benefit was not statistically definitive (77). Together, these studies indicate that pharmacodynamic immune activation alone is insufficient to ensure durable clinical benefit.

The limitations of this strategy are both biological and regimen dependent. ICB is unlikely to overcome resistance dominated by inadequate antigen-specific T-cell responses, myeloid suppression, or stromal exclusion, while overlapping hepatic, pulmonary, and hematologic toxicities may constrain concurrent administration. Not all resistant tumors therefore fail because of inadequate checkpoint signaling, and alternative strategies may be required when the dominant limitation lies in antigen priming. Comparative efficacy, immune correlates, and safety profiles are summarized in **Table 2**, with additional study details provided in **Supplementary Table 1**.

**Table 2:** Completed clinical studies of CDK4/6 inhibitor-based immunotherapeutic combinations.

Trial	Phase/regimen	Patient population	Clinical outcome	Immune/biomarker correlates	Major toxicities	Key message	References
Palbociclib-pembrolizumab (NCT02778685)	Phase I/II; palbociclib + pembrolizumab + letrozole	Newly diagnosed HR+/HER2− metastatic breast cancer	Clinical responses were observed, but confirmation in randomized studies is required.	Higher baseline effector-memory CD4^+^/CD8^+^ T cells were associated with response; increased peripheral cDC1 maturation; no predictive value for PD-L1, TILs, or TMB.	Grade 3-4 neutropenia, leukopenia, liver enzyme elevation	Demonstrated immune activation without validated predictive biomarkers.	(43, 109)
Abemaciclib-pembrolizumab (NCT02779751)	Phase Ib; abemaciclib + pembrolizumab ± anastrozole	CDK4/6 inhibitor-naïve HR+/HER2− metastatic breast cancer	Antitumor activity observed, but benefit-risk profile did not support further development.	No validated immune biomarker; exploratory PD-L1 and tissue analyses.	Severe hepatotoxicity, ILD/pneumonitis, two treatment-related deaths	Combination efficacy was offset by unacceptable toxicity.	(17)
NEWFLAME (jRCT2080224706)	Phase II; abemaciclib + nivolumab + endocrine therapy	First-/second-line HR+/HER2− metastatic breast cancer	Responses observed; trial terminated early because of toxicity.	Hepatotoxicity associated with CD8^+^ liver infiltration, increased inflammatory cytokines, and reduced peripheral Tregs.	Grade ≥3 neutropenia, ALT elevation, one ILD-related death	Immune activation may accompany severe immune-mediated toxicity.	(18)
CheckMate 7A8 (NCT04075604)	Phase Ib/II; nivolumab + palbociclib + anastrozole	Untreated ER+/HER2− primary breast cancer	Trial closed before randomized phase; efficacy could not be established.	No mature predictive immune biomarker identified.	Grade 3-4 hepatotoxicity, treatment discontinuation, pneumonitis	Toxicity prevented definitive evaluation of efficacy and biomarkers.	(110)
Ribociclib-spartalizumab (NCT03294694)	Phase I; ribociclib + spartalizumab ± fulvestrant	HR+/HER2− metastatic breast cancer and advanced ovarian cancer	Limited clinical activity despite pharmacodynamic immune effects.	Increased PD-1 occupancy and circulating activated CD8^+^ T cells; PD-L1, TILs, and TMB were not predictive.	Hypertransaminasemia, hematologic toxicity	Pharmacodynamic immune changes did not translate into meaningful clinical benefit.	(19)
PACE (NCT03147287)	Phase II; fulvestrant ± palbociclib ± avelumab	HR+/HER2− metastatic breast cancer after CDK4/6 inhibitor progression	Palbociclib continuation did not improve PFS; avelumab triplet showed only a numerical trend.	No validated immune biomarker; serial ctDNA and genomic analyses were exploratory.	Hematologic toxicity; immune-related adverse events	Did not support routine CDK4/6 inhibitor continuation after progression.	(77, 98)
MORPHEUS HR-positive breast cancer (NCT03280563)	Phase Ib/II umbrella platform; fulvestrant ± atezolizumab ± abemaciclib	HR+/HER2− advanced breast cancer after prior CDK4/6 inhibitor therapy	No definitive efficacy advantage for checkpoint-containing regimens.	Paired biopsies enabled exploratory immune profiling, but no validated predictive biomarker emerged.	Acceptable but regimen-dependent safety profile	Demonstrated feasibility of biomarker-rich platform trials rather than treatment selection.	(ClinicalTrials.gov NCT03280563)

Clinical efficacy endpoints and immune correlates should be interpreted separately. Most immune analyses were exploratory and were not powered to validate predictive biomarkers. Differences in disease setting, treatment duration, biopsy timing, analytical platform, and cohort composition limit direct cross-trial comparison.

cDC1, conventional type 1 dendritic cell; CD4, cluster of differentiation 4; CD8, cluster of differentiation 8; CDK4/6, cyclin-dependent kinase 4 and 6; DNA, deoxyribonucleic acid; ER, estrogen receptor; ESR1, estrogen receptor 1; HER2, human epidermal growth factor receptor 2; HR, hormone receptor; ILD, interstitial lung disease; NCT, ClinicalTrials.gov identifier; PD-1, programmed cell death protein 1; PD-L1, programmed death-ligand 1; PIK3CA, phosphatidylinositol-4,5-bisphosphate 3-kinase catalytic subunit alpha; TIL, tumor-infiltrating lymphocyte; TMB, tumor mutational burden.

### 6.3 Cancer vaccines and antigen-directed priming

Cancer vaccines may be particularly relevant when CDK4/6 inhibition preserves antigen presentation but endogenous T-cell priming remains insufficient. By increasing MHC-I expression, broadening the immunopeptidome, and activating interferon- or viral-mimicry programs, CDK4/6 inhibitors provide a biological rationale for exploiting a transient window of antigen-specific priming. Dendritic-cell vaccines have induced antigen-specific immune responses in selected breast-cancer settings, although these studies were not performed in combination with CDK4/6 inhibitors (78). Moreover, an expanded immunopeptidome does not necessarily indicate that the presented antigens are immunogenic, tumor specific, or shared across metastatic lesions. Vaccine-based combinations should therefore be regarded as mechanistically supported strategies for tumors with preserved antigen presentation but defective priming rather than established therapeutic sequences.

### 6.4 Adoptive cellular therapies

Adoptive cellular therapies may be most relevant when inadequate effector-cell quantity or function represents the dominant immune deficit. Although CDK4/6 inhibition can enhance antigen presentation and increase tumor-cell susceptibility to immune attack, it may also suppress activated lymphocyte proliferation, making treatment sequence an important consideration. A reported case of mutation-reactive tumor-infiltrating lymphocyte therapy demonstrated durable regression in metastatic HR-positive breast cancer, and early CAR-T-cell studies have shown feasibility in breast cancer, although neither established a CDK4/6-specific interaction (79, 80). A CDK4/6 inhibitor lead-in followed by washout before cell infusion is therefore biologically plausible, but whether this strategy improves cell trafficking, persistence, or antitumor efficacy remains unknown.

### 6.5 Bispecific antibodies and T-cell-engaging strategies

Bispecific antibodies may be most applicable when functional T cells are present but endogenous tumor recognition remains inefficient. CD3-based bispecific antibodies redirect endogenous T cells toward tumor-associated surface antigens, but their efficacy depends on adequate T-cell infiltration and stable antigen expression. Early anti-CD3×HER2 studies demonstrated biological feasibility in breast cancer without evaluating combination with CDK4/6 inhibitors (81). Antigen heterogeneity, T-cell exclusion, on-target/off-tumor toxicity, cytokine-release syndrome, and limited penetration into solid tumors remain important barriers. Consequently, these approaches are more likely to benefit selected immune-infiltrated tumors than to serve as broadly applicable partners for CDK4/6 inhibition.

### 6.6 Macrophage- and myeloid-directed immunotherapy

Myeloid-directed immunotherapy may be most appropriate when macrophage- or MDSC-dominant suppression represents the principal barrier to antitumor immunity. Candidate strategies aim to reduce myeloid recruitment, reprogram suppressive macrophage states, or enhance phagocytic activity, with intervention guided by functional state rather than macrophage abundance alone. Preclinical studies have linked CDK4/6 inhibitor resistance to immature myeloid-cell accumulation (47), a senescent fibroblast-IGF1/FGF7-STAT3/ARG1 macrophage circuit responsive to CSF1R inhibition (48), and enhanced antitumor activity following macrophage reprogramming combined with CD47 blockade (49). CXCR1/2-dependent stromal signaling may further promote myeloid recruitment (58), whereas IL25-positive macrophage states appear to represent a more restricted subtype (51). Collectively, these findings support selective myeloid reprogramming rather than indiscriminate macrophage depletion. Because tissue-resident macrophages also contribute to antigen presentation, tissue repair, and host defense, therapeutic strategies should aim to restore productive immune function rather than eliminate the myeloid compartment.

### 6.7 STING, TLR, and other innate-immune agonists

When defective innate sensing rather than suppressive myeloid states represents the dominant immune deficit, STING, TLR, and other innate-immune agonists may provide an alternative strategy for enhancing immune activation. STING agonists promote type I interferon production, dendritic-cell activation, cross-presentation, and chemokine expression, whereas TLR agonists provide an alternative route to enhance antigen priming (82–84). However, CDK4/6-associated viral-mimicry signaling should not be considered equivalent to STING activation because RNA- and DNA-sensing pathways engage distinct molecular mechanisms. Moreover, the activity of STING or TLR agonists depends on intact innate immune competence, while excessive activation may induce systemic inflammation, compensatory immune suppression, or myeloid remodeling (84, 85). Current support for combining innate-immune agonists with CDK4/6 inhibitors therefore remains derived primarily from preclinical or cross-tumor studies.

### 6.8 Cytokine and cytokine-pathway therapies

Cytokine-directed strategies may be particularly relevant when suppressive cytokine networks sustain myeloid accumulation, stromal remodeling, or chronic immune dysfunction during CDK4/6 inhibitor treatment. Depending on the dominant biological state, therapeutic approaches may involve enhancing effector-cell activity or inhibiting suppressive pathways, including TGF-β, IL-6, IL-8/CXCR1/2, CCL2/CCR2, CSF1/CSF1R, and senescence-associated secretory programs (14, 48, 57, 58). Because these pathways also contribute to tissue homeostasis and host defense, cytokine modulation may produce systemic toxicity or compensatory adaptation. Therapeutic decisions should therefore be guided by evidence of pathway activation within tumor tissue rather than isolated circulating cytokine concentrations.

### 6.9 Comparative translational framework

The strategies discussed above address distinct biological deficits and should be regarded as complementary rather than interchangeable. Their integration with CDK4/6 inhibitors depends on matching impaired antigen priming, deficient effector-cell function, defective innate sensing, myeloid suppression, stromal exclusion, or dysregulated cytokine signaling to an appropriate intervention. **Table 3** summarizes the corresponding immune states, evidence levels, principal limitations, and potential biomarkers for each strategy.

**Table 3:** Immunotherapeutic strategies beyond PD-1/PD-L1 blockade in the context of CDK4/6 inhibition.

Strategy	Representative modality	Candidate immune context	Current evidence	Candidate biomarker	Major toxicity	Key limitation	Ref.
Cancer vaccine	Peptide, RNA/DNA, viral or neoantigen vaccine	Antigen presentation preserved but insufficient priming	Early clinical	HLA, immunopeptidome, antigen-specific TCR	Autoimmune inflammation	Tumor heterogeneity; manufacturing	(21, 78)
Dendritic-cell vaccine	Antigen-loaded autologous DC	Defective T-cell priming	Early clinical	DC activation, antigen-specific ELISPOT/TCR	Local/systemic inflammation	Personalized manufacture	(78)
TIL therapy	Expanded mutation-reactive TILs	Existing tumor-reactive T cells	Early clinical	TIL fitness, neoantigen reactivity	Lymphodepletion, IL-2 toxicity	Low TIL yield; exhaustion	(79)
CAR-T/TCR-T	CAR-T or TCR-engineered T cells	Stable target antigen	Early clinical	Target density, HLA expression	CRS; neurotoxicity	Antigen loss; poor trafficking	(80)
CD3 bispecific antibodies	CD3 × tumor-antigen engager	Tumor-infiltrating T cells present	Early clinical	Target-antigen density	CRS; on-target toxicity	Antigen escape	(81)
NK/CAR-NK	NK or engineered NK cells	NK-accessible tumors	Preclinical	NK persistence; stress ligands	Cytokine-related toxicity	Limited persistence	(42, 81)
Dual checkpoint blockade	PD-1/CTLA-4 or PD-1/TGF-β blockade	Inflamed but immunosuppressed tumors	Cross-cancer clinical	PD-L1, checkpoint activity	Immune-related adverse events	Narrow therapeutic window	(17–20)
STING agonists	Intratumoral or systemic agonists	STING-competent, poorly primed tumors	Preclinical	STING signaling, type I IFN	Systemic inflammation	Pathway silencing; delivery challenges	(4)
TLR agonists	TLR3, TLR7/8 or TLR9 agonists	Defective innate activation	Preclinical	TLR activation; DC maturation	Cytokine release	Route- and formulation-dependent activity	(20)
Myeloid targeting	CSF1R, CCR2/CCL2, CXCR1/2 inhibitors	TAM/MDSC-dominant tumors	Preclinical	CSF1, CCL2, TAM/MDSC markers	Infection; hepatotoxicity	Myeloid heterogeneity	(14, 15, 48–51)
Phagocytosis modulation	CD47-SIRPα blockade	Macrophage-restricted phagocytosis	Preclinical	CD47, macrophage phenotype	Cytopenia	Broad normal-cell expression	(49)
Cytokine agonists	IL-2, IL-15, IL-12 or IFN agonists	Poor effector-cell expansion	Cross-cancer clinical	T/NK expansion	Vascular leak; systemic toxicity	Narrow therapeutic window	(20)
Cytokine/SASP blockade	TGF-β, IL-6, IL-8, CCL2 or CSF1 inhibition	Stromal/myeloid-dominant resistance	Preclinical	Pathway activation	Infection; impaired tissue repair	Pathway redundancy	(14–16, 37, 48, 57)

Current evidence reflects the highest level of supporting evidence available for each strategy. Most approaches remain investigational and require prospective biomarker validation before clinical implementation.

CAR, chimeric antigen receptor; CRS, cytokine-release syndrome; DC, dendritic cell; HLA, human leukocyte antigen; NK, natural killer; TIL, tumor-infiltrating lymphocyte.

Across all approaches, the major priorities remain biologically informed patient selection, rational treatment sequencing, longitudinal pharmacodynamic assessment, and evaluation of lineage-specific toxicity. Most strategies beyond ICB and selected myeloid-directed approaches are currently supported primarily by indirect or preclinical evidence and should be developed as prospective mechanistic hypotheses rather than empirical combinations or current treatment recommendations. Because resistant tumors are shaped not only by immune remodeling but also by metabolic adaptation, complementary therapeutic opportunities may emerge from targeting ferroptosis-related and nano-enabled vulnerabilities, which are discussed in the following section.

## 7 Ferroptosis and nano-enabled strategies for metabolic-immune reprogramming

### 7.1 Direct ferroptosis-related liabilities in CDK4/6 inhibitor resistance

The most direct evidence linking ferroptosis to CDK4/6 inhibitor resistance comes from ER-positive breast-cancer models in which GPX4 emerged from genome-wide CRISPR screening as a determinant of sensitivity to palbociclib and endocrine therapy. Genetic or pharmacological GPX4 perturbation increased sensitivity to palbociclib, giredestrant, and their combination, while treatment-associated oxidative stress and altered lipid metabolism produced an AGPAT3-dependent ferroptosis-sensitive state (82). A complementary luminal A study linked CDK4/6 inhibition to reduced SLC7A11 expression through impaired SP1 binding and showed that further SP1 or SLC7A11 interference enhanced activity in cell and animal models (83). Metastatic HR-positive/HER2-negative models provide additional evidence that targeting ferroptosis resistance can resensitize cells to palbociclib-endocrine therapy (84). Together, these studies identify candidate GPX4-system Xc− liabilities in selected preclinical models, although a clinically validated therapeutic window has not been established.

Additional endocrine-resistant and triple-negative breast-cancer models implicate other GPX4-regulatory pathways and demonstrate heterogeneity in ferroptosis states, but these findings are not specific to CDK4/6 inhibitor resistance (85–87). **Table 4A** therefore distinguishes direct biological-target evidence from broader non-nano mechanistic comparators.

**Table 4A:** Emerging ferroptosis- and nanomedicine-based strategies: biological and delivery characteristics.

Strategy/platform	Carrier class	Payload/mechanism	Targeting or delivery principle	Route	Disease/model	PK/biodistribution evidence	Developmental maturity	Ref
GPX4 sensitization	Non-nano comparator	GPX4 perturbation with palbociclib/giredestrant	None; systemic experimental intervention	Research/systemic	ER-positive cells and xenografts	Not applicable	Mechanistic preclinical	(82)
SLC7A11 targeting	Non-nano comparator	SP1/SLC7A11 interference with CDK4/6 inhibition	Transporter/pathway directed	Research/systemic	Luminal A cells and animal models	Not applicable	Mechanistic preclinical	(83)
RelB-GPX4 resistance	Non-nano comparator	RelB or GPX4 suppression	Pathway directed	Research/systemic	Tamoxifen-resistant breast-cancer models	Not applicable	Mechanistic preclinical	(85)
GPX4 inhibitor plus ICB	Non-nano comparator	GPX4 inhibition plus anti-PD-1	Biomarker-context hypothesis	Systemic	TNBC preclinical models	Not applicable	Mechanistic preclinical	(86)
Injectable SF platform	Protein hydrogel/local depot	GPX4-targeted ferroptosis-autophagy cargo	Local retention; stimulus-responsive release	Intratumoral/local	TNBC preclinical models	Partial; local distribution, incomplete systemic PK	Proof-of-concept preclinical	91
Magnetic composite nanoparticle	Inorganic/polymeric composite	Chemotherapy plus ferroptosis enhancement	Magnetic guidance/response	Systemic; magnetic intervention	TNBC preclinical models	Partial; platform-specific distribution	Proof-of-concept preclinical	92
Oral lipidic nanoplatform	Lipid-based carrier	Docetaxel/atorvastatin metronomic cargo	Transporter-directed oral delivery	Oral	TNBC PK and animal models	Partial-to-comprehensive within reported model	Advanced preclinical	95
Copper/erastin nanoparticle	Metal-containing core-shell carrier	Ferroptosis/cuproptosis induction plus ICB	Responsive co-delivery; no universal active ligand	Systemic	Colon and TNBC models	Limited/partial	Proof-of-concept preclinical	93
Palbociclib mesoporous polydopamine	Polymeric/inorganic hybrid	Palbociclib with immunogenic/photothermal functions	Stimulus/photothermal delivery	Systemic plus local energy	Breast-cancer preclinical model	Limited/partial	Proof-of-concept preclinical	96
Zero-valent-iron nanoparticle	Metal-containing nanoparticle	NRF2-associated ferroptosis/redox modulation	Passive/systemic delivery	Systemic	Lung-cancer models	Partial; non-breast evidence	Contextual preclinical	94

Strategies are grouped according to their predominant delivery platform or biological mechanism. Developmental maturity reflects the current stage of preclinical or translational development rather than clinical readiness.

GPX4, glutathione peroxidase 4; ICB, immune checkpoint blockade; NRF2, nuclear factor erythroid 2-related factor 2; PD-1, programmed cell death protein 1; PK, pharmacokinetics; SF, silk fibroin; SLC7A11, solute carrier family 7 member 11; SP1, specificity protein 1; TNBC, triple-negative breast cancer.

### 7.2 Lineage-specific redox consequences

A potential interferon-ferroptosis bridge is supported by experimental evidence showing that CD8-positive T-cell-derived interferon-γ can restrict SLC3A2/SLC7A11-dependent cystine uptake, while arachidonic acid availability can promote ACSL4-dependent tumor-cell ferroptosis (75, 76). These findings support an immune-ferroptotic connection in experimental systems, but this relationship has not been demonstrated directly in patients with CDK4/6 inhibitor-resistant disease.

Redox effects are lineage specific. Lipid uptake and lipid peroxidation can impair intratumoral CD8-positive T-cell function, extracellular GPX4 can alter dendritic-cell activity, and ferroptosis-resistant neutrophils may support immunosuppressive metastatic behavior (88–90). Thus, interventions designed to increase tumor-cell lipid peroxidation may also injure effector or antigen-presenting immune cells. The therapeutic goal is to enhance tumor-cell ferroptotic susceptibility while preserving immune-cell fitness and normal-tissue redox reserve. An interferon-high state alone is insufficient to infer ferroptosis sensitivity.

### 7.3 Nano-enabled selectivity and current evidence boundaries

These lineage-specific redox effects make nano-enabled delivery relevant only when it improves the selectivity and therapeutic index of ferroptosis-inducing interventions. Because tumor and immune-cell populations may differ in redox vulnerability, meaningful evaluation requires intracellular payload release, pharmacodynamic target engagement, and preservation of immune-cell function rather than nanoparticle accumulation alone.

Nano-enabled ferroptosis platforms have shown proof-of-concept activity in breast-cancer models (91, 92), while cross-tumor studies provide additional evidence that nanodelivery can couple ferroptosis induction with immune reprogramming (93–95). A mesoporous-polydopamine platform delivering palbociclib provides a related example of nano-enabled CDK4/6 inhibitor immunotherapy, although it did not establish ferroptosis-mediated reversal of CDK4/6 inhibitor resistance (96). None of these platforms has been validated as a resistance-reversal strategy in patients receiving CDK4/6 inhibitors.

Nano-enabled ferroptosis therapy should therefore be regarded as a delivery strategy for a biologically defined vulnerability rather than an independent resistance mechanism. Its translational value will depend on reproducible formulation, controlled payload release, pharmacodynamic target engagement, lineage-specific safety, and demonstrable improvement in therapeutic index. **Table 4B** compares the chemistry, manufacturing, and controls, scale-up, safety, and regulatory-readiness considerations of representative platforms. Ultimately, translating these therapeutic concepts into clinical practice will require biomarker frameworks capable of identifying the relevant biological states, monitoring pharmacodynamic responses, and guiding longitudinal patient stratification, as discussed in the following section.

**Table 4B:** CMC, safety, and regulatory readiness of representative nano-enabled platforms.

Platform	Key CMC challenge	Scale-up challenge	Safety concern	Translational readiness	Key requirement	Representative references
Injectable SF platform	Hydrogel composition, drug loading, release consistency	Sterile manufacture; reproducible gelation	Local inflammation; incomplete degradation	Preclinical proof-of-concept	PK, repeat-dose toxicology, CMC validation	(91)
Magnetic composite nanoparticle	Particle size, magnetic properties, drug loading	Batch uniformity; magnetic-process reproducibility	Metal retention; liver/spleen accumulation	Preclinical	Biodistribution, long-term safety, potency assay	(92)
Oral lipidic nanoplatform	Lipid stability; ligand density; drug leakage	Oral formulation and storage stability	GI toxicity; hepatic uptake; immune effects	Advanced preclinical	Human PK, chronic toxicology, comparability	(95)
Copper/erastin nanoparticle	Copper valence; drug loading; ion release	Multicomponent synthesis; storage stability	Copper toxicity; off-target oxidative injury	Proof-of-concept	Dose window, bioanalysis, persistence studies	(93)
Palbociclib mesoporous polydopamine	Drug loading; pore-size control; coating consistency	Photothermal reproducibility	Thermal injury; particle persistence	Early preclinical	Thermal dosimetry, sterility, biodistribution	(96)
Zero-valent iron nanoparticle	Iron oxidation state; surface chemistry	Oxidation during manufacture and storage	Iron overload; oxidative injury	Contextual preclinical evidence	Breast-cancer validation; long-term safety	(94)

The listed considerations summarize representative chemistry, manufacturing, and control (CMC), pharmacological, and translational issues reported for each platform. They are intended as a comparative framework rather than a formal regulatory evaluation.

CMC, chemistry, manufacturing, and control; GI, gastrointestinal; NRF2, nuclear factor erythroid 2-related factor 2; PK, pharmacokinetics.

## 8 Candidate biomarker-informed framework for longitudinal evaluation

### 8.1 Molecular and metabolic resistance context

Molecular profiling provides the biological context within which immune remodeling should be interpreted. Tissue and plasma analyses can identify resistance mechanisms involving RB1, CCNE1/CCNE2-CDK2, CDK6, ESR1, PIK3CA, PTEN, and related pathways, with interpretation strengthened when baseline tissue, serial plasma, and progression samples are available (5, 6, 31, 32, 35). Serial ctDNA can further track clonal evolution and may detect molecular progression before radiographic progression. Early on-treatment ctDNA dynamics and post-progression mutational profiling have demonstrated prognostic value in HR-positive/HER2-negative breast cancer (97, 98).

The strongest clinical evidence currently supports longitudinal monitoring of emerging ESR1 mutations. The PADA-1 study demonstrated that treatment adaptation based on rising ESR1 mutations detected by ctDNA before radiographic progression is feasible, and companion analyses confirmed the robustness of droplet digital PCR and next-generation sequencing for mutation detection (99, 100). More recently, SERENA-6 extended this strategy by evaluating ctDNA-guided switching to camizestrant in the first-line setting (101, 102). Molecular evolution may also be accompanied by metabolic adaptation. OXPHOS-, redox-, and ferroptosis-related measurements may therefore provide additional biological context, although these dimensions reflect distinct biological states and should not be treated as interchangeable biomarkers (7).

### 8.2 Tissue, spatial, and multimodal immune profiling

Tissue-based analysis defines the local immune and stromal ecosystem, while spatial profiling reveals immune exclusion, disrupted tumor-lymphocyte contact, and cellular neighborhoods that are not captured by density measurements alone (41, 53–56, 61–65). Circulating biomarkers provide a complementary view of systemic tumor evolution and immune adaptation (43–45). Whereas ctDNA primarily reflects tumor burden and clonal dynamics, circulating cellular, soluble, and T-cell clonal signals may indicate systemic immune activation or suppression during treatment. Tissue and blood measurements should therefore be regarded as complementary rather than interchangeable and interpreted through longitudinal sampling.

High-dimensional profiling further resolves the cellular origin, functional state, and spatial organization of resistant tumor, immune, and stromal populations that cannot be separated by bulk measurements alone. Recent computational frameworks integrating multimodal single-cell, spatial, digital pathology, and clinical data provide complementary approaches for identifying candidate resistance states and composite biomarker signatures (103–105). These integrated approaches are intended to generate biologically interpretable hypotheses rather than clinically validated predictive algorithms.

### 8.3 Longitudinal sampling and prospective validation

**Figure 3:**
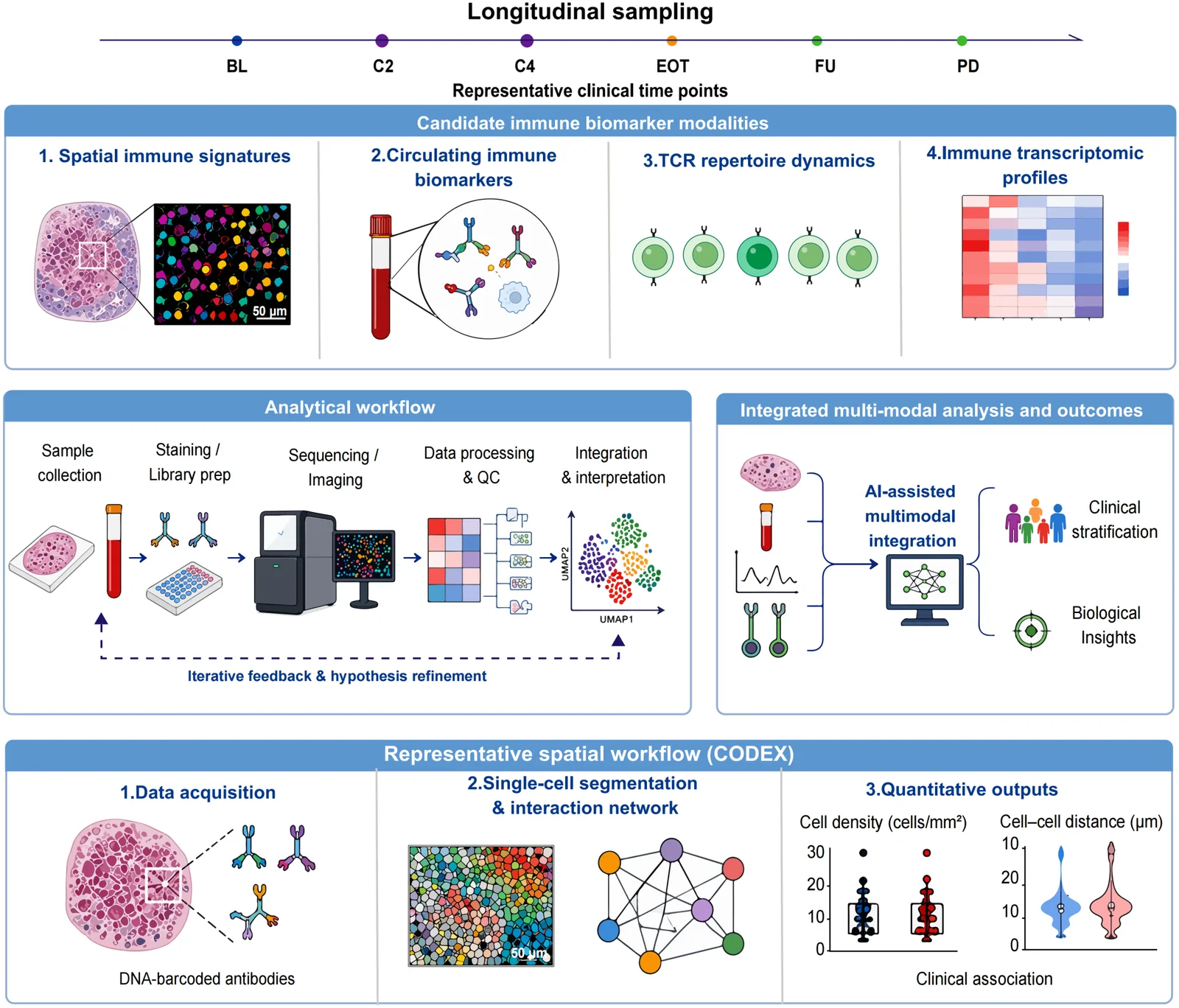
Candidate longitudinal framework for multimodal immune-biomarker assessment during CDK4/6 inhibitor-based therapy. Longitudinal sampling and candidate biomarker modalities. Biospecimens may be collected at representative clinical time points, including baseline (BL), on-treatment (C2 and C4), end of treatment (EOT), follow-up (FU), and progressive disease (PD). The proposed framework integrates four complementary biomarker domains: spatial immune signatures, circulating immune biomarkers, T-cell receptor (TCR) repertoire dynamics, and immune transcriptomic profiles. Analytical workflow. Tissue and blood samples undergo staining, library preparation, sequencing or imaging, followed by standardized data processing, quality control, and multimodal integration to characterize longitudinal immune remodeling. The feedback loop indicates iterative refinement of sampling strategies and biological hypotheses. Integrated multimodal analysis. Spatial, circulating, repertoire, transcriptomic, and clinical data are integrated using computational approaches, including AI-assisted analysis, to support exploratory patient stratification and improve understanding of treatment response, residual disease, and resistance evolution. This framework is intended for prospective biomarker development rather than clinical decision-making. Representative spatial workflow. CODEX is shown as an example of high-dimensional spatial imaging. DNA-barcoded antibodies enable multiplex tissue profiling, followed by image acquisition, single-cell segmentation, cell-cell interaction analysis, and quantitative assessment of spatial features such as immune-cell density and cellular proximity, which can subsequently be correlated with clinical outcomes.

Because CDK4/6 inhibitor resistance evolves dynamically, biomarker assessment should extend beyond a single pretreatment specimen. **Figure 3** illustrates a longitudinal framework incorporating baseline, early on-treatment, end-of-treatment, follow-up, and progression sampling. Biomarker analyses from neoadjuvant palbociclib-based studies further support the value of serial tissue sampling for characterizing pharmacodynamic responses, although these findings remain exploratory and require prospective validation (106). Sampling timing and analytical depth should therefore be matched to the underlying biological question rather than applied as a universal protocol (**Table 5**).

**Table 5:** Candidate biomarkers for longitudinal evaluation of CDK4/6 inhibitor response and immune adaptation.

Time point	Biomarker domain	Representative biomarkers	Method	Biological significance	Potential application	Major limitation	Representative references
Baseline	Genomic resistance	RB1, ESR1, PIK3CA/PTEN, CCNE1/2, CDK6	NGS; ddPCR	Defines molecular resistance background	Baseline stratification	Does not predict immune state	(5, 6, 32, 67)
	Immune composition	CD8, FOXP3, CD68/CD163, CD11c, PD-L1, HLA-I, FAP	IHC; mIF	Baseline immune and stromal context	Cohort characterization	Marker-dependent variability	(9, 10, 30, 43)
	Spatial organization	CD8-Treg, CD8-TAM, CAF-TAM neighborhoods	mIF; spatial profiling	Immune exclusion versus inflamed phenotype	Spatial biomarker development	No standardized cutoff	(10, 30, 41)
	Immune transcriptome	IFN, antigen-presentation, T-cell, myeloid, CAF signatures	RNA-seq; NanoString	Baseline immune programs	Stratification	Cell-composition confounding	(9, 12, 30)
	Metabolic/redox state	OXPHOS, GPX4, SLC7A11, ROS, OCR	RNA/protein; metabolomics	Candidate ferroptosis susceptibility	Exploratory biomarker	Functional dependence unresolved	(7, 82–84)
Early on-treatment	Pharmacodynamics	pRB, Ki-67, Cyclin E	IHC	Target engagement	Early PD assessment	Timing dependent	(9, 106)
	IFN/antigen presentation	HLA-I, IFN-response signatures	RNA-seq; mIF	Early immune priming	Pharmacodynamic endpoint	Transient changes	(4, 9, 12)
	Circulating immune cells	Tregs, MDSCs, cDC1	Flow cytometry	Systemic immune remodeling	Tissue-blood comparison	Blood ≠ tumor	(43–45)
	Cytokines/chemokines	CXCL9/10, CCL2, IL-6/8/17A	ELISA; multiplex assay	Systemic inflammatory signals	Exploratory PD marker	Multiple biological sources	(14–18)
	ctDNA dynamics	Variant allele frequency	NGS; ddPCR	Early molecular response	Molecular monitoring	Not immune-specific	(6, 97–102)
	TCR dynamics	Clonality, expansion	Bulk/scTCR-seq	Adaptive immune response	Longitudinal tracking	Tumor specificity uncertain	(23, 24)
Residual disease	T-cell state	Granzyme B, PD-1, TIM-3, LAG-3	mIF; scRNA-seq	Activation versus dysfunction	Residual-state classification	Function cannot be inferred from abundance	(10, 11, 13)
	Myeloid state	MDSCs, ARG1, CSF1R, SPP1	mIF; scRNA-seq	Myeloid adaptation	Mechanistic studies	Heterogeneous phenotypes	(10, 15, 47–49)
	CAF/ECM	FAP, α-SMA, collagen, periostin, p16/p21	Spatial profiling	Stromal barrier formation	Stromal biomarker development	CAF heterogeneity	(37–39, 57–65)
Progression	Acquired resistance	Emerging RB1, ESR1, PI3K alterations	NGS; ddPCR	Clonal evolution	Trial re-stratification	Lesion heterogeneity	(5, 6, 98–102)
	Immune-stromal niche	T cells, TAMs, CAFs, checkpoints	Spatial profiling; scRNA-seq	Resistant ecosystem	Mechanistic reclassification	Repeat biopsy feasibility	(10, 11, 30)
Longitudinal integration	Multimodal state	Genomic, spatial, transcriptomic, TCR, metabolic features	AI/ML integration	Composite resistance state	Exploratory prediction model	External validation required	(103, 104)

The listed biomarkers represent candidate biomarkers proposed for longitudinal biomarker development rather than validated biomarkers for clinical decision-making. Suggested sampling windows are intended as a conceptual framework for prospective translational studies.

CAF, cancer-associated fibroblast; cDC1, conventional type 1 dendritic cell; CITE-seq, cellular indexing of transcriptomes and epitopes by sequencing; ctDNA, circulating tumor DNA; ddPCR, droplet digital PCR; ECM, extracellular matrix; FFPE, formalin-fixed paraffin-embedded; IHC, immunohistochemistry; mIF, multiplex immunofluorescence; mIHC, multiplex immunohistochemistry; MDSC, myeloid-derived suppressor cell; NGS, next-generation sequencing; PBMC, peripheral blood mononuclear cell; SASP, senescence-associated secretory phenotype; TCR, T-cell receptor.

Across molecular, tissue, circulating, spatial, and multimodal domains, major limitations include intratumoral heterogeneity, incomplete tissue-blood concordance, variable tumor shedding, limited serial sampling, assay heterogeneity, and the absence of prospectively validated thresholds. Clinical development will require prespecified hypotheses, harmonized sampling and analytical pipelines, reproducible measurements, independent multicenter validation, and clinically interpretable end points before routine clinical implementation.

## 9 Challenges and future perspectives

Despite substantial progress in understanding how CDK4/6 inhibitors reshape the breast cancer immune ecosystem, translating these biological insights into clinically effective therapeutic strategies remains challenging. Most mechanistic evidence is derived from experimental models that differ substantially from human HR-positive/HER2-negative breast cancer in immune composition, tumor evolution, treatment history, and drug exposure. Although these models remain essential for establishing causal mechanisms, their findings require validation in well-designed clinical cohorts before informing patient care (107, 108).

Current evidence indicates that CDK4/6 inhibitor resistance is not a single biological entity but comprises multiple resistance states with distinct molecular, immune, stromal, and metabolic characteristics. Future studies should therefore prioritize defining biologically coherent and clinically reproducible resistance states rather than continually expanding the number of candidate biomarkers.

Key questions remain unresolved, including which immune alterations are transient or persistent, which arise from tumor-intrinsic versus microenvironmental adaptation, and which resistance states remain therapeutically reversible. Addressing these questions will require integrated molecular, immune, spatial, and longitudinal analyses to establish clinically interpretable resistance states.

Ultimately, the goal is to develop biologically informed therapeutic strategies rather than increasingly complex biomarker panels. As resistance states become more reproducible and clinically actionable, state-informed combination therapies and patient stratification may enable more rational management of CDK4/6 inhibitor-resistant HR-positive/HER2-negative breast cancer.

## 10 Conclusion

CDK4/6 inhibitors have transformed the treatment of HR+/HER2− breast cancer, yet their biological effects extend well beyond cell-cycle arrest. Accumulating evidence indicates that these agents dynamically remodel the tumor immune ecosystem through coordinated changes in tumor cells, immune populations, stromal compartments, and metabolic programs. Rather than representing a uniform transition from immune activation to immune suppression, resistance emerges through heterogeneous and evolving biological states shaped by interactions between tumor-intrinsic mechanisms and the tumor microenvironment.

This Review highlights a state-informed perspective that complements established molecular mechanisms of resistance by integrating molecular alterations with immune, stromal, spatial, and metabolic remodeling. Within this framework, therapeutic vulnerability is determined by the evolving resistance ecosystem rather than by individual molecular alterations alone. Accordingly, biologically matched therapeutic strategies, longitudinal tissue-blood evaluation, and integrated biomarker assessment may provide a more rational basis for future translational studies than empirical combination approaches.

Future progress will depend on longitudinal human studies, harmonized biomarker platforms, and prospective clinical trials capable of linking biologically defined resistance states to therapeutic response. As these resistance states become more reproducible and clinically interpretable, state-informed therapeutic strategies may enable more precise management of patients with CDK4/6 inhibitor-resistant HR+/HER2− breast cancer.
