# Supplementary material for: Cyclin-dependent kinase 4 and 6 inhibitors and the breast cancer immune ecosystem: immune remodeling, resistance, and therapeutic reprogramming
Source: Front Immunol. 2026 Aug 21;17:1923129. doi: 10.3389/fimmu.2026.1923129 (PMC13542978; doi:10.3389/fimmu.2026.1923129)
Supplement: Supplementary file 1 [file Table1.docx]

**Supplementary Material**

**Table S1. Detailed design, sampling, biomarker methods, and outcomes of completed CDK4/6 inhibitor–based immunotherapy studies.**

| **Strategy category** | **Trial / identifier** | **Phase / design** | **Patient population** | **Regimen / sequence** | **Primary endpoint** | **Efficacy / current results** | **Immune correlates / planned analyses** | **Biomarkers, specimens and sampling** | **Major toxicities** | **Status** | **Key message / limitation / reference** |
| --- | --- | --- | --- | --- | --- | --- | --- | --- | --- | --- | --- |
| A. Standard endocrine combinations and sequencing | PALOMA-2 NCT01740427 | Phase III; randomized, double-blind | Previously untreated postmenopausal ER-positive/HER2-negative advanced breast cancer | Palbociclib plus letrozole versus placebo plus letrozole | Investigator-assessed progression-free survival | Palbociclib prolonged progression-free survival versus letrozole alone. | NR; the pivotal trial was not designed for longitudinal immune profiling. | Tumor biomarkers in exploratory analyses; immune assays NR | Expected myelosuppression, dominated by neutropenia; febrile neutropenia was uncommon. | Completed; published | [CE] Establishes efficacy benchmark, not an immune-predictive biomarker. (117) |
| A. Standard endocrine combinations and sequencing | MONALEESA-2 NCT01958021 | Phase III; randomized, double-blind | Previously untreated postmenopausal HR-positive/HER2-negative advanced breast cancer | Ribociclib plus letrozole versus placebo plus letrozole | Investigator-assessed progression-free survival | Ribociclib improved progression-free and overall survival. | NR in the pivotal efficacy analysis. | Tumor RNA/genomic correlatives in secondary studies; immune assays not prespecified for clinical selection | Neutropenia was common; liver-function and corrected-QT monitoring were required. | Completed; published | [CE] Defines standard efficacy and regimen-specific safety context. (118, 119) |
| A. Standard endocrine combinations and sequencing | MONARCH 3 NCT02246621 | Phase III; randomized, double-blind | Previously untreated postmenopausal HR-positive/HER2-negative advanced breast cancer | Abemaciclib plus anastrozole or letrozole versus placebo plus anastrozole or letrozole | Investigator-assessed progression-free survival | Abemaciclib improved progression-free survival and objective response. | NR in the pivotal clinical report. | Routine tumor assessment; immune assays NR | Diarrhea, neutropenia and leukopenia were the principal toxicities. | Active, not recruiting; primary results published | [CE] Provides an efficacy comparator for experimental immune combinations. (120) |
| A. Standard endocrine combinations and sequencing | SONIA NCT03425838 | Phase III; randomized strategy trial | HR-positive/HER2-negative advanced breast cancer without prior advanced-disease therapy | First-line versus second-line use of a CDK4/6 inhibitor with sequential endocrine therapy | Progression-free survival after two treatment lines | Early use did not significantly improve the primary two-line progression-free endpoint. | No validated immune correlate; correlative work focused on molecular evolution. | Serial plasma circulating tumor DNA and tumor genomics | First-line use increased cumulative CDK4/6 inhibitor exposure and grade 3 or higher adverse events. | Active, not recruiting; published | [NEG][BD] Supports prospective testing of treatment sequence and longitudinal biomarkers. (121, 122) |
| B. CDK4/6 inhibitor plus immune checkpoint blockade | Palbociclib–pembrolizumab study NCT02778685 | Phase I/II; single-arm cohorts | Newly diagnosed HR-positive/HER2-negative metastatic breast cancer | Palbociclib plus pembrolizumab plus letrozole | Safety, tolerability and objective response | Responses were observed, but the small nonrandomized study requires confirmation. | Higher baseline effector-memory CD4⁺/CD8⁺ subsets correlated with response; peripheral cDC1 maturation increased, and classical monocytes decreased. Baseline TILs, TMB, and PD-L1 were not associated with response. | Serial peripheral blood mononuclear-cell flow cytometry; tumor-infiltrating lymphocytes; PD-L1; tumor mutational burden | Frequent grade 3–4 neutropenia and leukopenia; liver-enzyme elevations occurred. | Active, not recruiting; results posted and published | [IP][BD] Generates circulating immune hypotheses without a validated cutoff. (43, 115) |
| B. CDK4/6 inhibitor plus immune checkpoint blockade | Abemaciclib–pembrolizumab phase 1b NCT02779751 | Phase Ib; nonrandomized cohorts | CDK4/6 inhibitor-naive HR-positive/HER2-negative metastatic breast cancer | Abemaciclib plus pembrolizumab with or without anastrozole | Safety and tolerability | Antitumor activity was observed, but the benefit–risk profile did not support further development. | No immune biomarker was validated for response prediction. | Tumor PD-L1 and exploratory tissue analyses; routine pharmacokinetics | Severe transaminase elevations, excess interstitial lung disease/pneumonitis and two treatment-related deaths. | Active, not recruiting; breast-cancer cohorts published | [SAF][NEG] Demonstrates that immune-combination safety is regimen specific. (17) |
| B. CDK4/6 inhibitor plus immune checkpoint blockade | NEWFLAME jRCT2080224706; UMIN000036970 | Phase II; multicohort, nonrandomized | First- or second-line HR-positive/HER2-negative metastatic breast cancer | Abemaciclib plus nivolumab plus fulvestrant or letrozole | Objective response rate | Responses occurred, but enrollment stopped early because of toxicity. | Hepatotoxicity was associated with CD8-positive liver infiltration, elevated tumor necrosis factor-related cytokines and interleukin-11, and reduced peripheral regulatory T cells. | Serial blood, tumor, and fecal sampling; cytokines; regulatory T cells; liver biopsy | Frequent grade 3 or higher neutropenia and alanine aminotransferase elevation; one death from interstitial lung disease. | Terminated early; published | [SAF][IP] Immune activation may accompany severe immune-mediated toxicity. (18) |
| B. CDK4/6 inhibitor plus immune checkpoint blockade | CheckMate 7A8 NCT04075604 | Phase Ib/II; neoadjuvant safety run-in | Untreated ER-positive/HER2-negative primary breast cancer | Nivolumab plus palbociclib plus anastrozole | Dose-limiting toxicity; planned residual-cancer-burden endpoint | The study closed before the randomized phase; efficacy could not be established. | No mature predictive immune correlate was established. | Pretreatment and on-treatment tumor sampling was planned; immune results were not definitive | High incidence of grade 3–4 hepatotoxicity and treatment discontinuation; pneumonitis occurred. | Completed early; published | [SAF][NEG] Safety prevented a definitive efficacy or biomarker test. (116) |
| B. CDK4/6 inhibitor plus immune checkpoint blockade | Ribociclib–spartalizumab study NCT03294694 | Phase I; dose escalation and expansion | HR-positive/HER2-negative metastatic breast cancer and advanced ovarian cancer | Ribociclib plus spartalizumab with or without fulvestrant | Maximum tolerated dose, recommended phase II dose and safety | Two confirmed partial responses occurred in the breast-cancer triplet cohort; efficacy was limited. | PD-1 occupancy and activated circulating CD8-positive T cells increased irrespective of response; PD-L1, tumor-infiltrating lymphocytes, and tumor mutational burden did not correlate with progression-free survival. | Peripheral-blood flow cytometry; serum thymidine kinase; PD-L1; tumor-infiltrating lymphocytes; tumor mutational burden; next-generation sequencing | Marked hypertransaminasemia and hematologic toxicity limited development. | Terminated; published | [IP][SAF][NEG] Pharmacodynamic immune change did not establish clinical benefit. (19) |
| B. CDK4/6 inhibitor plus immune checkpoint blockade | PACE NCT03147287 | Phase II; randomized | HR-positive/HER2-negative metastatic breast cancer progressing on a CDK4/6 inhibitor plus an aromatase inhibitor | Fulvestrant versus fulvestrant plus palbociclib versus fulvestrant plus palbociclib plus avelumab | Progression-free survival for fulvestrant versus fulvestrant plus palbociclib | Continuing palbociclib did not improve progression-free survival; the avelumab triplet showed a nondefinitive numerical signal. | No validated immune predictor; the avelumab comparison was not powered to establish an immune-selected benefit. | Tumor and plasma genomics, including ESR1 and PIK3CA; serial circulating tumor DNA | Expected hematologic toxicity; checkpoint-related events require regimen-specific interpretation. | Active, not recruiting; primary and circulating-tumor-DNA analyses published | [NEG][BD] Supports randomized confirmation and resistance-context profiling. (80, 98) |
| B. CDK4/6 inhibitor plus immune checkpoint blockade | MORPHEUS HR-positive breast cancer NCT03280563 | Phase Ib/II; randomized umbrella platform | HR-positive/HER2-negative advanced breast cancer after progression on prior CDK4/6 inhibitor therapy | Fulvestrant with or without atezolizumab and an abemaciclib–fulvestrant–atezolizumab triplet among platform arms | Objective response rate | Platform results did not establish a definitive efficacy advantage for checkpoint-containing therapy. | Mandatory on-treatment biopsies were incorporated, but no validated immune predictor has been reported. | Paired tumor biopsy; PD-L1 and exploratory molecular/immune profiling | Combination safety was monitored across small platform cohorts; interpretation is arm specific. | Completed; registry results and conference report available | [BD][NEG] Illustrates biopsy-enabled testing but not a treatment-selection rule. |
| C. CDK4/6/endocrine therapy versus chemotherapy-based strategies | KENDO NCT03227328 | Phase II; randomized, open-label | Aggressive HR-positive/HER2-negative metastatic breast cancer | Investigator-selected CDK4/6 inhibitor plus endocrine therapy versus chemotherapy plus endocrine therapy | Progression-free survival | The prematurely closed study showed no chemotherapy superiority and numerically favored CDK4/6 inhibition plus endocrine therapy. | Immune-related gene signatures, tumor-infiltrating lymphocytes and tertiary lymphoid structures showed favorable exploratory trends in the CDK4/6 inhibitor arm. | PAM50; tumor RNA profiling; tumor-infiltrating lymphocytes; tertiary lymphoid structures | No new class-wide safety signal; the small sample limits comparison. | Completed; published | [CE][BD] Connects molecular subtype and immune context to comparative strategy testing. (123) |
| D. CDK4/6 inhibitor plus targeted therapy | TRINITI-1 NCT02732119 | Phase I/II; single-arm | Endocrine-refractory HR-positive/HER2-negative advanced breast cancer after progression on a CDK4/6 inhibitor | Ribociclib plus everolimus plus exemestane | Dose finding and 24-week clinical benefit rate | The triplet met its prespecified clinical-benefit benchmark in a nonrandomized cohort. | NR; biomarker analyses focused on resistance-pathway alterations. | Tumor/plasma genomic alterations, including ESR1 and PIK3CA | Neutropenia and stomatitis were common; no new safety signal was identified. | Completed; published | [CE][BD] Supports pathway-directed investigation after progression on a CDK4/6 inhibitor. (124) |
| D. CDK4/6 inhibitor plus targeted therapy | INAVO120 NCT04191499 | Phase II/III; randomized, double-blind | Endocrine-resistant, PIK3CA-mutated HR-positive/HER2-negative advanced breast cancer | Inavolisib plus palbociclib plus fulvestrant versus placebo plus palbociclib plus fulvestrant | Progression-free survival | The inavolisib triplet substantially improved progression-free survival. | NR; the validated enrichment variable was PIK3CA mutation, not immune state. | PIK3CA mutation in tissue or plasma; circulating tumor DNA | Neutropenia remained common; hyperglycemia, stomatitis and diarrhea increased with inavolisib. | Active, not recruiting; primary and overall-survival results published | [CE][BD] Demonstrates molecular enrichment without validating an immune biomarker. (125, 126) |
| D. CDK4/6 inhibitor plus targeted therapy | VIKTORIA-1 NCT05501886 | Phase III; randomized | HR-positive/HER2-negative advanced breast cancer after CDK4/6 inhibitor and aromatase inhibitor therapy | Gedatolisib plus fulvestrant with or without palbociclib versus standard therapy | Progression-free survival by PIK3CA stratum | In PIK3CA-wild-type disease, gedatolisib combinations improved progression-free survival versus fulvestrant. | NR; molecular strata rather than immune states defined the analysis. | PIK3CA mutation status; tumor/plasma molecular profiling | Triplet therapy increased neutropenia; stomatitis, rash, hyperglycemia and diarrhea were monitored. | Active, not recruiting; PIK3CA-wild-type results published | [CE][BD] Supports resistance-pathway stratification after prior CDK4/6 inhibition. (127) |
| E. Neoadjuvant and window-of-opportunity studies | neoMONARCH NCT02441946 | Phase II; randomized lead-in/window study | Postmenopausal HR-positive/HER2-negative stage I–IIIB breast cancer | Abemaciclib, anastrozole or both during a two-week lead-in, followed by combination therapy | Change in tumor Ki-67 at two weeks | Abemaciclib-containing arms produced marked Ki-67 suppression and cell-cycle arrest. | Combination therapy increased cytokine signaling, antigen-presentation and activated-T-cell transcriptional programs. | Serial tumor biopsy; Ki-67 immunohistochemistry; bulk RNA profiling | Diarrhea, constipation and nausea predominated; toxicity was generally manageable. | Completed; published | [IP][BD] Supports an early, tissue-based immune-pharmacodynamic window. (9) |
| E. Neoadjuvant and window-of-opportunity studies | CORALLEEN NCT03248427 | Phase II; randomized, open-label | Postmenopausal PAM50 luminal-B/HER2-negative operable breast cancer | Ribociclib plus letrozole versus multiagent chemotherapy | PAM50 low risk-of-relapse status at surgery | Ribociclib plus letrozole produced a low-risk-of-relapse conversion rate similar to chemotherapy. | Serial transcriptomic analyses showed treatment- and time-dependent immune-cell and antigen-presenting-cell programs rather than a uniform durable activation. | Baseline, day-15 and surgical tissue; PAM50; risk-of-relapse score; RNA profiling | Distinct endocrine/CDK4/6 and chemotherapy toxicity profiles limit direct comparison. | Completed; published | [IP][BD] Links serial tissue biology to a chemotherapy-sparing strategy hypothesis. (12, 128) |
| E. Neoadjuvant and window-of-opportunity studies | PALLET NCT02296801 | Phase II; randomized window study | Postmenopausal ER-positive primary breast cancer | Letrozole with immediate or delayed palbociclib versus letrozole alone | Ki-67 change and clinical response at 14 weeks | Palbociclib enhanced Ki-67 suppression but did not improve short-term clinical response. | NR; the study primarily measured proliferation and apoptosis. | Serial core biopsy; Ki-67 and cleaved-poly(ADP-ribose)-polymerase immunohistochemistry | Grade 3 or higher toxicity increased, mainly asymptomatic neutropenia. | Completed; published | [IP][NEG] Strong pharmacodynamic arrest did not translate into greater short-term clinical response. (129) |
| E. Neoadjuvant and window-of-opportunity studies | RIBECCA NCT03096847 | Phase IIIb; single-arm | HR-positive/HER2-negative locally advanced or metastatic breast cancer | Ribociclib plus letrozole | Clinical benefit at 24 weeks | Clinical activity was accompanied by measurable peripheral immune changes. | Adaptive-immune signatures increased and regulatory T-cell frequency decreased; effector/memory populations and T-cell receptor diversity were largely stable, with clonality increases in selected patients. | Serial peripheral blood mononuclear cells; flow cytometry; immune-gene expression; T-cell receptor sequencing | Consistent with known ribociclib toxicity; the immune analysis was exploratory. | Completed; immune-correlative analysis published | [IP][BD] Shows peripheral immune modulation without universal clonal expansion. (44) |
| E. Neoadjuvant and window-of-opportunity studies | NeoRHEA NCT03065621 | Phase II; single-arm neoadjuvant biomarker study | ER-positive/HER2-negative early breast cancer | Palbociclib plus endocrine therapy for four cycles | Baseline RNA biomarkers of resistance | The study defined pharmacodynamic and resistance-associated states rather than a comparative efficacy benefit. | Treatment decreased T-cell proliferation and CD8-positive/CD103-positive tissue-resident-memory features in paired tumor samples. | Paired tumor RNA and chromatin profiling; multiplex immunohistochemistry; personalized circulating tumor DNA | Consistent with neoadjuvant palbociclib plus endocrine therapy. | Completed; immune and circulating-tumor-DNA analyses published | [IP][BD][NEG] Demonstrates that sustained treatment can attenuate selected adaptive-immune features. (13, 130) |
| E. Neoadjuvant and window-of-opportunity studies | SAFIA NCT03447132 | Phase III; randomized, double-blind | Operable low-recurrence-score luminal/HER2-negative breast cancer responding to induction endocrine therapy | Fulvestrant with or without goserelin followed by palbociclib or placebo | Pathologic complete response | Adding palbociclib did not improve pathologic complete response. | NR; immune profiling was not a defining endpoint. | 21-gene recurrence score for enrollment; pathologic response | Expected palbociclib-related hematologic toxicity; no immune-specific signal reported. | Completed; published | [NEG][BD] Biomarker selection by recurrence score did not establish added pathologic benefit. (131) |
| F. Ongoing or biomarker-driven intervention studies | PADA-1 NCT03079011 | Phase III; randomized, open-label | ER-positive/HER2-negative advanced breast cancer receiving first-line aromatase inhibitor plus palbociclib | Circulating-ESR1-mutation-triggered switch to fulvestrant plus palbociclib versus continuation of aromatase inhibitor plus palbociclib | Progression-free survival after molecular randomization and hematologic safety | Switching endocrine therapy at rising ESR1 mutation before radiologic progression improved progression-free survival. | NR; the intervention was based on a molecular resistance marker. | Serial plasma circulating tumor DNA; droplet digital PCR with next-generation-sequencing cross-validation | Hematologic toxicity was consistent with continued palbociclib. | Completed; published | [CE][BD] Validates a specific ESR1 monitoring strategy, not a universal resistance rule. (99, 100) |
| F. Ongoing or biomarker-driven intervention studies | SERENA-6 NCT04964934 | Phase III; randomized, double-blind | ER-positive/HER2-negative advanced breast cancer with emerging ESR1 mutation during first-line aromatase inhibitor plus CDK4/6 inhibitor | Switch to camizestrant with continuation of the CDK4/6 inhibitor versus continuation of the aromatase inhibitor combination | Progression-free survival | Switching at molecular emergence of ESR1 mutation prolonged progression-free survival. | NR; the tested trigger was ESR1 mutation in circulating tumor DNA. | Serial plasma circulating tumor DNA every two to three months | Low discontinuation rates; regimen-specific ocular, cardiac and hematologic monitoring applied. | Active, not recruiting; primary and extended analyses published | [CE][BD] Confirms prospective value of a narrowly specified molecular trigger. (101, 102) |

Note: The table preserves the complete study-level information contained in the original manuscript table. Direct immunotherapy combinations are presented together with contextual endocrine, targeted, window-of-opportunity, and biomarker studies retained from the original clinical evidence synthesis. “Not reported” indicates that the original manuscript table did not provide the field.

Abbreviations: BD, biomarker discovery; CE, clinical efficacy; IP, immune pharmacodynamics; NEG, negative or nondefinitive result; SAF, safety signal. Abbreviations: AKT, protein kinase B; ALT, alanine aminotransferase; AST, aspartate aminotransferase; cDC1, conventional type 1 dendritic cell; CD4, cluster of differentiation 4; CD8, cluster of differentiation 8; CDK4/6, cyclin-dependent kinase 4 and 6; ctDNA, circulating tumor DNA; DNA, deoxyribonucleic acid; ER, estrogen receptor; ESR1, estrogen receptor 1; HER2, human epidermal growth factor receptor 2; HLA, human leukocyte antigen; HR, hormone receptor; IHC, immunohistochemistry; IL, interleukin; ILD, interstitial lung disease; MDSC, myeloid-derived suppressor cell; MHC-I, major histocompatibility complex class I; NCT, ClinicalTrials.gov identifier; NGS, next-generation sequencing; NR, not reported; ORR, objective response rate; OS, overall survival; PAM50, prediction analysis of microarray 50; PBMC, peripheral blood mononuclear cell; PCR, polymerase chain reaction; PD-1, programmed cell death protein 1; PD-L1, programmed death-ligand 1; PEPI, preoperative endocrine prognostic index; PFS, progression-free survival; PIK3CA, phosphatidylinositol-4,5-bisphosphate 3-kinase catalytic subunit alpha; RNA, ribonucleic acid; RNA-seq, RNA sequencing; ROR, risk of recurrence; TCR, T-cell receptor; TIL, tumor-infiltrating lymphocyte; TMB, tumor mutational burden; Treg, regulatory T cell.

**Table S2. Ongoing and recruiting clinical studies of CDK4/6 inhibitor–based immunotherapeutic strategies.**

| **Strategy category** | **Trial / identifier** | **Phase / design** | **Patient population** | **Regimen / sequence** | **Primary endpoint** | **Efficacy / current results** | **Immune correlates / planned analyses** | **Biomarkers, specimens and sampling** | **Major toxicities** | **Status** | **Key message / limitation / reference** |
| --- | --- | --- | --- | --- | --- | --- | --- | --- | --- | --- | --- |
| D. CDK4/6 inhibitor plus targeted therapy | CAPItello-292 NCT04862663 | Phase Ib/III; randomized phase III | HR-positive/HER2-negative advanced or metastatic breast cancer | Capivasertib plus fulvestrant and investigator-selected palbociclib, ribociclib or abemaciclib versus fulvestrant plus a CDK4/6 inhibitor | Dose-limiting toxicity and progression-free survival | Mature phase III efficacy results are not yet available. | NR; no validated immune correlate is available. | Tumor/plasma AKT-pathway alterations and circulating tumor DNA | Ongoing assessment of overlapping metabolic, dermatologic, gastrointestinal and hematologic toxicity. | Recruiting | [BD] Tests pathway co-targeting; biomarker and safety definitions remain prospective. |
| F. Ongoing or biomarker-driven intervention studies | SIGNAL-ER 101 NCT07214532 | Interventional; single-group, biomarker-guided | HR-positive/HER2-negative early breast cancer after standard local and endocrine therapy | Tumor-informed circulating-tumor-DNA surveillance with protocol-directed CDK4/6 inhibitor initiation | Invasive disease-free survival | No efficacy results are available. | NR; immune and spatial correlates are not yet validated. | Serial plasma tumor-informed circulating tumor DNA; optional translational specimens | Safety will reflect the selected CDK4/6 inhibitor and endocrine backbone. | Recruiting | [BD] Prospectively tests molecular surveillance; it does not yet validate a treatment trigger. |

Note: Information for ongoing and recruiting studies was extracted from publicly available clinical trial registries and corresponding publications, where available. Recruitment status reflects the information available at the time of manuscript preparation and may change as trials progress. Immune-correlative analyses, biomarker assessments, and translational endpoints listed for ongoing studies represent planned or exploratory objectives and should not be interpreted as validated predictive biomarkers or treatment-guiding strategies.

Abbreviations: AKT, protein kinase B; CDK4/6, cyclin-dependent kinase 4 and 6; ctDNA, circulating tumor DNA; DNA, deoxyribonucleic acid; ER, estrogen receptor; HER2, human epidermal growth factor receptor 2; HR, hormone receptor; NCT, ClinicalTrials.gov identifier; NR, not reported.
